# Bioprinting Vasculature: Materials, Cells and Emergent Techniques

**DOI:** 10.3390/ma12172701

**Published:** 2019-08-23

**Authors:** Clarissa Tomasina, Tristan Bodet, Carlos Mota, Lorenzo Moroni, Sandra Camarero-Espinosa

**Affiliations:** MERLN Institute for Technology-inspired Regenerative Medicine, Complex Tissue Regeneration Department, Maastricht University, P.O. Box 616, 6200MD Maastricht, The Netherlands

**Keywords:** bioprinting, vascularization, tissue engineering

## Abstract

Despite the great advances that the tissue engineering field has experienced over the last two decades, the amount of in vitro engineered tissues that have reached a stage of clinical trial is limited. While many challenges are still to be overcome, the lack of vascularization represents a major milestone if tissues bigger than approximately 200 µm are to be transplanted. Cell survival and homeostasis is to a large extent conditioned by the oxygen and nutrient transport (as well as waste removal) by blood vessels on their proximity and spontaneous vascularization in vivo is a relatively slow process, leading all together to necrosis of implanted tissues. Thus, in vitro vascularization appears to be a requirement for the advancement of the field. One of the main approaches to this end is the formation of vascular templates that will develop in vitro together with the targeted engineered tissue. Bioprinting, a fast and reliable method for the deposition of cells and materials on a precise manner, appears as an excellent fabrication technique. In this review, we provide a comprehensive background to the fields of vascularization and bioprinting, providing details on the current strategies, cell sources, materials and outcomes of these studies.

## 1. Introduction

In an aging society, our quality of life appears to be dependent on our ability to regenerate damaged or diseased tissues that are otherwise unable to self-heal or regenerate into functional organs. Thus, tissue engineering has emerged as a potential solution to this end [1]. Based on the combination of cells and materials to create de novo tissues that can replace the damaged areas or induce the regeneration of the native ones, tissue engineering has seen an evolution in many aspects such as the range of materials and biofabrication techniques used or the capability to mimic complex tissues. Despite this increasing knowledge in tissue engineering strategies and our ability to generate relatively large micro-tissues in vitro, the field is facing an important challenge which resolution will determine the success or failure of the same one; the vascularization of engineered tissues [2,3,4].

Most of the tissues in the body are vascularized and their proper functioning relies on the diffusion of nutrients and oxygen, and waste removal from the surrounding blood vessels. Indeed, the diffusion limit of oxygen is said to be of 100–200 µm, depending on the tissue, and the cell survival above this limit appears to be compromised [5]. In vitro, nutrient supply and oxygen diffusion in avascular tissues of more than 200 µm is possible via different engineering techniques such as artificial perfusion. However, in vivo implantation of large in vitro generated tissue or tissue-scaffold constructs fails if vascularization of the construct is not achieved [2].

Upon in vivo implantation of cell-scaffold constructs, vascularization starts, partly as a result of an inflammatory wound healing response and partly due to the secretion of angiogenic growth factors from the implanted cells that are subjected to an hypoxic environment due to the absence of vasculature [6]. However, spontaneous vascularization follows the native process of vessel formation, which is limited to few tenths of micrometers per day, resulting on an implanted construct that is non-vascularized for up to several weeks depending on the size of the same one [7]. While spontaneous vascularization might be sufficient when thin tissues such as skin or other avascular ones such as cartilage are targeted, complications arise on larger defects where gradients in oxygen and nutrient diffusion have been measured [8]. This is particularly important to tissues such as bone were the critical size defects are usually of several centimeters in length [9,10].

Strategies to promote vascularization of non-vascularized tissue engineered constructs have been focused on: (i) the design of scaffolds with specific porosities that allow for the inclusion of in vivo formed new vessels; (ii) the delivery of exogenous angiogenic growth factors, cytokines or cells from implant materials in vivo; or (iii) the surface immobilization of peptide sequences or full proteins that promote in vivo angiogenesis [11,12]. Pre-vascularization in vitro has relied heavily on the self-organization capability of endothelial cells (ECs) and co-cultures of this to form vascular networks [13,14,15]. However, the fabrication of vascularized complex tissues via bottom-up strategies such as bioprinting appear to be more appealing, allowing for the precise patterning of cells and materials in a three-dimensional (3D) environment [16,17,18,19]. Bioprinting of pre-vascularized engineered tissues or organ bioprinting is however an emerging technique that still requires optimization of the different components of the deposited bioink. Cell sources, materials, scaffold design and bioprinting approaches have to be orchestrated for each of the specific targeted tissues and play a crucial role on the bioprinting process that ultimately determines the success of the construct.

In this review, we present the background as well as latest advances on bioprinting of vascularized tissues considering cell sources, materials, vasculature design and bioprinting techniques used.

## 2. Structure and Composition of the Vascular System

Vessels and vascular networks are responsible for transport of blood throughout the human body to the different tissues. This system’s function is to carry nutrients, gasses and chemical waste to guarantee the correct cell function. In recent years, significant progress has been achieved in creating bioprinted vascularized networks for tissue engineering. The successful development and clinical application of these constructs requires a deep understanding of the mechanisms, the cells and molecules involved in healthy and pathological vascular conditions.

### 2.1. Blood Vessel Composition and Properties

Blood vessels constitute a branched and intricate system throughout our body to conduct blood from and to our tissues and organs. According to location and specific function, the structure of the blood vessels can highly specialize and differ in structure and mechanical properties to meet particular needs. The blood vessels are structurally organized in three concentric layers or tunics, namely the *intima* or endothelium, the tunica *media* and the *adventitia*, each one varying from the others on the matrix composition and the cells the reside within [20]. The *intima* is the innermost layer composed by a monolayer of ECs that is in direct contact with the blood flow. This layer, also called endothelium, acts as a barrier between the vessel and the blood stream to prevent bacterial infiltrations and thrombus formation. Surrounding the *intima*, the basement membrane of collagen IV and laminin is flexible and perfusable, allowing substances to pass through it. Furthermore, an internal elastic lamina, mostly present in large arteries, is found at the boundary with the next layer to enhance elasticity. The middle layer is the tunica *media* and is composed by smooth muscle cells (SMCs) embed in a matrix of collagen (type I and II), proteoglycans and elastin. Along the vessel axis, SMCs and collagen fibers arrange in a helically oriented pattern. Surrounding the tunica *media*, the external elastic lamina provides elasticity and structural support. Finally, the outermost cellular layer is the *adventitia*, formed by fibroblast supported by a loosely arranged collagen matrix whose function is to stabilize the tissue and prevent tearing.

**Figure 1:**
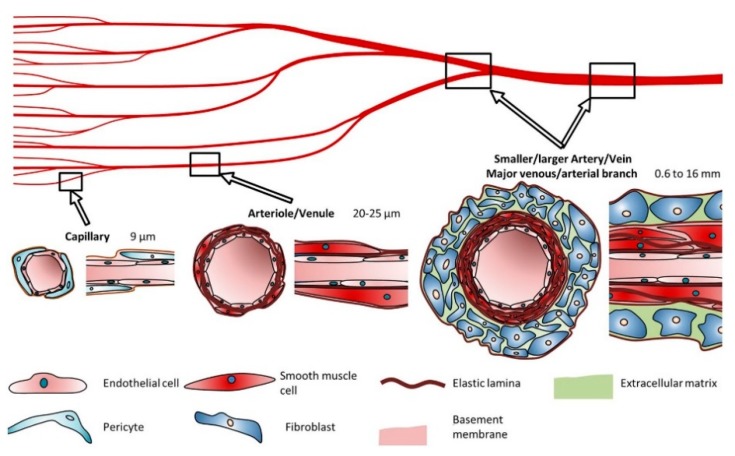
Blood vessels cell types and composition with diameter range. Reprinted by permission from Springer Nature: Nature, Scientific Reports, Reference [21], © 2018.

The number of layers and their thickness depends on the physiological function that the vessels perform (Figure 1). Capillaries have a diameter of 9 µm and are formed by just a single layer of ECs surrounded by pericytes to allow a rapid and efficient diffusion of oxygen and nutrients, while arteries (0.6–16 mm diameter range) have thick flexible walls able to hold the high pressure and continuous diameter changes they are subjected to. On the other hand, veins with a 1–15 mm diameter sustain low blood pressures so they deform easily and have a thin-wall that lacks the distinct structural organization of arteries [21].

The extracellular matrix (ECM) in the blood vessels is the key factor to their native mechanical properties [22]. Characteristics such as tensile stiffness, elasticity, compressibility and viscoelasticity are vital for the correct lifelong functioning of the tissue. Tensile stresses are higher in the *adventitia* of human coronary arteries with values of 1430 ± 604 kPa circumferential and 1300 ± 692 kPa longitudinal tensile stress, which are given by the collagen, mainly type I and III in the layers while the proteoglycans contribute to compressibility [23]. Elasticity is provided by elastin fibers while viscoelasticity by collagen and elastin together. In human carotid arteries, static and dynamic distensibility were measured under mean arterial pressure of 68–112 mmHg and resulted in 2.85 ± 1.05  ×  10^−5^ Pa^−1^ and 1.57 ± 0.67  ×  10^−5^ Pa^−1^, respectively, while the viscosity phase angle is ~0.1 at 2 Hz [24].

### 2.2. Vasculogenesis, Angiogenesis and Remodeling

To design and create appropriate bioprinted vascular networks, the mechanisms dictating vascular formation and remodeling have to be considered. The two main processes in vascular formation are vasculogenesis and angiogenesis. Vasculogenesis refers to the de novo formation of blood vessels during embryogenesis [25]. Since nutrient transport is crucial for embryonic survival and healthy growth, the first functional organ system to be developed is the cardiovascular system. During vasculogenesis, embryonic vascular precursors stem cells called angioblasts migrate from the mesoderm and form blood islands. In these structures, they aggregate, proliferate and differentiate into endothelial progenitors cells (EPCs). EPCs then start to organize onto tubular structures and to form primitive microcapillaries. In adults, vasculogenesis can also occur during tissue healing or tumor outgrow [26]. Angiogenic growth factors are secreted from cells in the blood and mobilize a unique cell population of the bone marrow of EPCs [27]. These cells are able to travel through the blood circulation and reach the desired location to proliferate and differentiate into mature ECs. Hence, they come together to form a new vascular network to supply blood.

Angiogenesis refers to the formation of new vessels from preexisting vascular networks. Lack of oxygen and nutrient in tissues trigger the ECs to break out from their stable position and start branching to reach the hypoxic site until oxygen levels are restored. New vessels can form by sprouting from pre-existing ones (sprouting angiogenesis) or by splitting from an origin (non-sprouting angiogenesis) [2,28]. Sprouting angiogenesis occurs when the vessel is destabilized and its ECM is proteolytically degraded, allowing EC migration and proliferation. The broken ECM offers a temporary support for cellular processes until the lumen is fully formed. On the other hand, during non-sprouting angiogenesis, ECs proliferate inside the vessel increasing the lumen size that can be split by transcapillary pillars.

During angiogenesis, a determining role is played by growth factors. These small molecules not only initiate angiogenesis, but are also involved in the regulation and remodeling of new blood vessels. Vascular endothelial growth factor (VEGF), basic fibroblast growth factor (bFGF) and platelet-derived growth factor (PDGF or CD31) are the main participants [29]. VEGF is produced by many cells and is a potent mitogen and chemoattractant to ECs. This angiogenic growth factor is a potent stimulator for ECs cellular processes as well as a shape regulator during sprouting angiogenesis. bFGF promotes the proliferation, migration and differentiation of ECs, while PDGF promotes maturation and remodeling.

After the formation of new capillaries by angiogenesis, a following process called arteriogenesis can eventually take place in newly collateral arteries [30]. Arteriogenesis is most likely caused by blood flow shear stresses and it involves the stabilization and remodeling of vessels that leads to the maturation of collateral bridges between arterial networks. Indeed, newly formed vessels become covered by mural cells recruited by PDGF, which give the vessel viscoelastic and vasomotor properties by creating a muscular sheath.

Remodeling is an active and adaptive process that occurs both during vasculogenesis and angiogenesis [31]. The cells of the endothelium strategically sense the long-term changes in the surrounding environment including growth modulators, vasoactive substances and inflammatory mediators circulating in the blood flow. In response to an alteration to the homeostatic balance, the cells adapt by making the vessel undergo structural modifications. First, the vessel wall perceives the changes in the hemodynamic conditions of the blood stream such as pressure and flow or even injury. Then, this recognition step is followed by intracellular communication throughout the different cell layers, which results in the synthesis and release of molecules. These substances entail changes in cell growth and death, cell migration and production or degradation of the ECM, which leads to the reconstruction and destruction of both cellular and acellular vessel components.

## 3. Materials for Bioprinting

Bioprinting is an additive manufacturing technique involving a hydrogel, also termed bioink, used to encapsulate cells or aggregates. Bioinks can be created from natural and synthetic biomaterials or a mixture of both (Table 1) [32]. These bioinks must fulfill different criteria to be suitable for this process [33]. Ideally, a bioink should not only match the biological and mechanical properties of the tissue, but also satisfy the bioprinting modalities. During or after the bioprinting process, bioink crosslinking should allow retaining of the final shape and structure and provide a support for cell viability and cellular processes. A bioink should possess the ideal rheological characteristics such as certain viscosity at a given shear rate and gelation capability in order to be processed [34]. The viscosity of the hydrogel should be tunable to allow homogenous distribution of the cells before bioprinting and fulfill the relevant range for the bioprinting technology (3.5–12 mPa·s for inkjet printing, 30–6 × 10^7^ mPa·s for extrusion printing, and 1–300 mPa·s for laser printing) [34]. As mentioned, another important parameter is the applied shear rate. Most polymer solutions display a shear thinning behavior which is a decrease in viscosity with the increase of shear rate. Furthermore, the shear rate influences also the cells encapsulated within the material and can led to cell aggregation or even cell death depending on shear forces. Last, after bioprinting, the bioink should recover and retain the imprinted shape. The fastest the bioink undergoes rapid gelation and solidifies to achieve stability, the higher the resolution would be in the final construct. A list highlighting the most commonly used bioinks and the different methods for bioprinting used is presented in Table 1.

### 3.1. Naturally-Derived Bioinks

Traditionally used natural-derived bioinks include agarose, alginate, collagen, fibrin, gelatin and hyaluronic acid. They have the intrinsic advantage of closely resembling the natural cell environment, because they possess similar properties or are components of the ECM itself [57]. However, they do not possess high mechanical properties, leaving the cells with a weak mechanical 3D environment.

Agarose is a linear polysaccharide constituted by repeating units of a disaccharide of d-galactose and 3,6-anhydro-l-galactopyranose. Properties such as biocompatibility, mechanical strength and gelation mechanism have made this biomaterial a choice for tissue engineering applications [58]. The gelation of agarose can occur without the need of crosslinker at low temperature (30–40 °C) and can melt at 80–95 °C. Thermo-reversible gelation occurs as temperature decreases and agarose chains rearrange into helices and further into a bundle network of random conformation [59]. In agarose, proliferation and attachment of ECs is inferior compared to other biomaterials such as fibrin or collagen [60]. To improve cell adhesion and spreading, agarose has been blended with other natural polymers such as collagen and fibrinogen [61]. Both blends were printable, but the collagen-agarose hydrogels showed an increase in the storage modulus compared to their single components. Degradation was not detected in any of the blends, but the fibrin-agarose hydrogels showed significant water uptake compared to the collagen-agarose ones. Co-culture of ECs and fibroblasts in collagen and agarose hydrogels for 14 days resulted in enhanced capillary formation, cell migration and shape stabilization as compared to fibrinogen and collagen hydrogels. Agarose has been used in scaffold-free bioprinting as a mold or support for cell aggregates [35]. In this study, agarose rods were printed as a molding template for multicellular vascular spheroids. Spheroids composed of Chinese hamster ovary cells (CHO), human umbilical vein smooth muscle cells (HUVSMCs), or human skin fibroblasts (HSFs) alone or in combination were generated using capillary micropipettes. During seven days of culture, agarose allowed spatial control of the vascular channel diameter and patterning and fusion of the spheroids was observed, which resulted in single- and double-layered small diameter vascular tubes.

**Figure 2:**
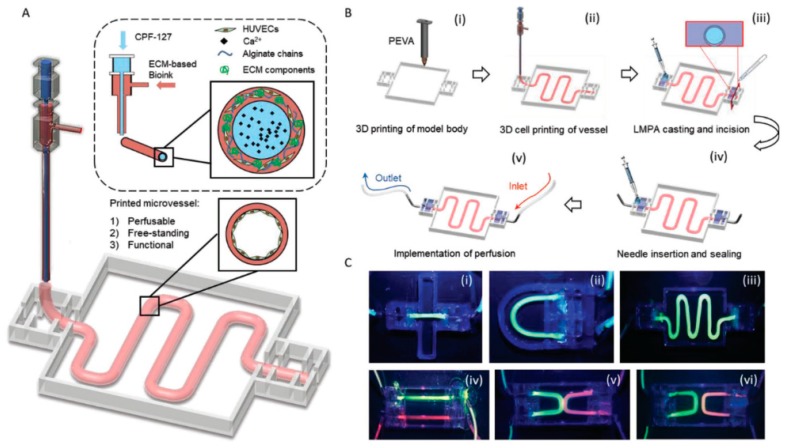
Bioprinting system for ECM-based bioink with a core of Pluronic and calcium ions and a shell of HUVECs encapsulated in alginate and ECM components (**A**). Different steps in the fabrication of the vascular model (**B**): (i) 3D printing of the PEVA model body; (ii) the 3D cell printing; (iii) casting and incision of low melting point agarose; (iv) needle insertion and sealing; and (v) perfusion. Perfusion of vessels construct with different designs ((i) straight; (ii) curved; (iii) serpentine; (iv) dual-parallel; (v) attached dual-curves; and (vi) discrete dual-curves) (**C**). Reprinted from Reference [37] with permission from John Wiley and Sons.

Alginate is a linear polysaccharide formed by units of β-(1,4) linked d-mannuronic acid and its epimer α-(1,4) linked l-guluronic acid. The carboxylic groups present in alginate permit the formation of ionic crosslinks via divalent cations, such as calcium. Ionic crosslinking is usually carried out in calcium chloride (CaCl_2_) or calcium sulfate (CaSO₄) solutions. Alginate is a widely used bioink in vascular application due to the broad range of different crosslinking and bioprinting methods that have been developed as well as its inherent biocompatibility [62,63]. Alginate has been used as bioink in vascular applications to build constructs with both horizontal and vertical bifurcations [36] and zigzag structures [64] using inkjet bioprinting. Furthermore, alginate was also used to bioprint human umbilical vein endothelial cells (HUVECs) using co-axial deposition systems [37]. The final zigzag tube was composed by a core of sacrificial Pluronic and calcium ions and by a shell of HUVECs encapsulated in alginate and ECM components (Figure 2). The alginate was rapidly crosslinked and the construct maintained the structural and functional integrity throughout culture and after removal of the pluronic. The HUVECs formed a mature monolayer endothelium with permeability and self-remodeling properties after seven days of culture.

Hydrogels can be formulated and bioprinted by blending alginate with other polymers such as polyethylene glycol monoacrylate-fibrinogen (PF) [65]. PF can be crosslinked post-printing by UV light irradiation and it is useful to provide a matrix for cell culture while the alginate is a sacrificial supportive hydrogel to better control fiber deposition. HUVECs and induced pluripotent cell-derived cardiomyocytes (iPSC-CMs) were extruded through a microfluidic bioprinting head (MPH) in a Janus cell pattern. After bioprinting, the alginate was washed using EDTA for a better iPSC culture. The cells displayed a vessel-like organization with lumen of about 150 μm. However, the final bioink was achieved by the introduction of 1 wt.% polyethylene glycol-diacrylate (PEG-DA) monomer within the PF/ALG bioink for the CMs in order to be able to modulate the stiffness and composition.

Collagen type I is the most abundant collagen in our body. It is fibrous and composed by three left-handed polyproline II-type helices that assemble into a right-handed bundle [66]. It is rich in heterotrimers of two α1 (I) and one α2 (I) chains and it provides tensile stiffness and load bearing in tissues [67]. This biomaterial is well known for its cell attachment and proliferation properties due to the presence of specific cell binding domains. However, gelation of collagen occurs slowly (requiring up to half an hour at 37 °C) resulting in nonhomogeneous cell distribution, which makes this bioink not easily compatible with bioprinting modalities. Collagen has been employed in combination with other bioinks as sacrificial template or as biopaper. Biopapers are flat biomimetic scaffolds fabricated through molding [68] or electrospinning [69], compatible with cell printing and stackable to form a 3D cell construct [70]. A bioprinted gelatin channel with encapsulated HUVECs was perfused into a 3 mm collagen sacrificial template scaffold [40]. After the gelatin removal, the construct kept the shape for up to three weeks of perfusion. The cells already extensively covered the surface in three days and on Day 5 a vascular endothelial cadherin (VE-cadherin) staining showed the adherent junctions. However, cells in dynamic culture looked elongated, straightened because of the flow, and did not proliferate into the collagen scaffold while cells in static culture did and formed angiogenic sprouts up to 400 µm on Day 7. The same group exploited this technique by depositing a fibrin gel containing HUVECs and fibroblasts within two perfusable sacrificial gelatin channels [71]. Fibrin and cells were deposited in between two gelatin channels, and then collagen was printed on top and gelatin was liquefied. HUVECs were injected in the gelatin channels and cells were cultured for 14 days by channel perfusion. Cell viability and angiogenic sprouting were achieved by applying gentle flow through the channels in culture. In another work, thyroid spheroids and allantoic spheroids containing thyrocytes and ECs were bioprinted within a collagen hydrogel [72]. It was possible to observe spheroid fusion during culture as well as the vascularization of the thyroid spheroid from the ECs in the allantoic spheroids. Furthermore, collagen I gel was also employed in bioprinting of human aortic smooth muscle cells (AoSMCs) and StemPro© cell tissue spheroids [73]. During culture, spheroids were able to fuse and form ring-like and tube-like vascular constructs but with an internal diameter reduction of 50% after spheroid fusion and tissue formation. In a recent study, ECs labeled with fluorescent protein dTomato were patterned via laser-assisted bioprinting onto a collagen biopaper with embedded mesenchymal stem cells (MSCs) [42]. High EC density allowed the development of a stable and connected network but the deposition of VEGF and hMSCs onto the collagen biopaper improved formation and stability.

Fibrin is a fibrous protein involved in blood clotting and wound healing [74]. Polymerization and gel formation are initiated by thrombin, a proteolytic enzyme that cleaves the fibrinogen peptides to obtain fibrin monomers that later assembled to form a fiber network. Fibrin networks are characterized by the presence of soft filaments which give the gel nonlinear elasticity [75]. In vascular tissue engineering applications, fibrin has been employed to construct in vitro capillaries using thermal inkjet printing [43]. Fibrin was used to print micro-sized channels for the culture of human microvascular endothelial cells (HMVECs), which maintained integrity and showed cell confluent lining at 21 days. However, even if disadvantages such as fast degradability and weak mechanical properties can be encountered, gelatin can still be employed as sacrificial material to support other polymers [21]. In this work, a drop-on-demand bioprinting system was used to construct an in vitro blood vessel model. The model consisted of the three blood vessel layers, mimicking the native structure of the *intima*, the *media* and the *adventitia* with endothelium, SMCs and fibroblast, respectively. The HUVECs were printed in a gelatin sacrificial material, the SMCs around the fibrinogen with an outer layer of thrombin while the surrounding ECM of fibroblasts was casted from a collagen solution. Cell viability after printing was high (>83%) and, during the culture, the expression of VE-Cadherin and CD31, smooth muscle actin, and collagen IV was observed.

Gelatin is the product of the denaturation and structural degradation of collagen. Physical gelation of gelatin is reversible and the gel is formed by the collagen structure assembly or crystallization at low temperatures [76]. Thermally-induced gelation is not the only way to obtain gelatin hydrogels, but also through chemical crosslinking with the addition of a crosslinker. Gelatin was employed to bioprint vascularized liver tissue [45]. Using a dual nozzle extrusion system, bioinks of a blend of gelatin, alginate and chitosan laded with hepatocytes and a blend of gelatin, alginate and fibrinogen with adipose derived stem cells (ADSCs) induced into endothelial-like cells were extruded. The addition of alginate to gelatin enabled a fast crosslinking. ADSCs cultures on these deposited structures were positive for CD31, a mature endothelial marker. However, even if the expression of CD31 was observed, few spindle shapes were present, and only at the periphery of the strands. Other strategies to facilitate a fast crosslinking and to increase the mechanical properties of gelatin-based hydrogels use methacrylated modified gelatin (GelMA). GelMA derives from denaturated collagen and it is composed by methacrylamide and methacrylate groups [77]. Under UV exposure, it can be crosslinked to form hydrogel with broadly tunable mechanical and physicochemical properties. Therefore, challenges have been encountered in developing bioprinting techniques with incorporated photocrosslinking [78]. In addition, GelMA cannot be successfully extruded at concentrations below 7% resulting in a high concentration hydrogel, which limits cell processes. In one study, mouse pre-osteoblasts cells were bioprinted in 10% GelMA and perfuse with HUVECs using a microchannel created with agarose [47]. The endothelial monolayer observed had high cell viability after bioprinting and provided mass transport over time. GelMA biological properties were also improved through the blending with natural biomaterials such as collagen I [79]. This bioink was used to encapsulate HUVECs and hMSC and the collagen addition upgraded cell spreading, capillary-like structure formation as well as printing properties.

Hyaluronic acid (HA) is present in the connective tissues of our body. It is a linear polysaccharide consisting of repeating units of β-1,4-d-glucuronic acid–β-1,3-*N*-acetyl-d–glucosamine. HA is highly biocompatible and it is degraded in the presence of hyaluronidases. HA exhibits low mechanical properties and slow gelation, which is why it is often chemically functionalized or blended with other polymers. Microscale continuous optical bioprinting (μCOB) allowed the rapid bioprinting of glycidal methacrylate-hyaluronic acid (GM-HA) and GelMA [50]. This particular polymer blend offered tunable mechanical properties, cell viability and photocrosslinking capability, overcoming the main issues encountered with neat gelatin-based hydrogels. HUVECs and HepG2 or 10T1/2 were bioprinted in prevascularized constructs. These networks were bioprinted in two different configurations: uniform channels or gradient channels (50–250 μm diameter range). HUVECs were bioprinted in the channels in GM-HA and gelatin methacrylate (GelMA), while the supportive cells HepG2 or 10T1/2 in the surroundings in GelMA. HUVECs and 10T1/2 displayed endothelial network formation in vitro and were sequentially implanted under the dorsal skin of SCID mice. Even if the hydrogel pattern was not maintained in vivo, CD31 and hVWF staining confirmed the formation of endothelial network. Other studied chemical modifications of gelatin include a methacrylated ethanolamide derivative of gelatin (GE-MA). This was crosslinked together with methacrylated hyaluronic acid (HA-MA) [51]. The blended hydrogel HA-MA:GE-MA was extrudable and was used to encapsulate fibroblasts NIH 3T3, hepatoma cells (HepG2 C3A), and human intestinal epithelial cells (Int 407). The amount of incorporated GE-MA was critical to achieve a gel with both adequate mechanical properties and adherent sites for cells. The bioink was deposited in the desired form and maintained the cells viable during seven days of culture.

### 3.2. Synthetic Bioinks

**Figure 3:**
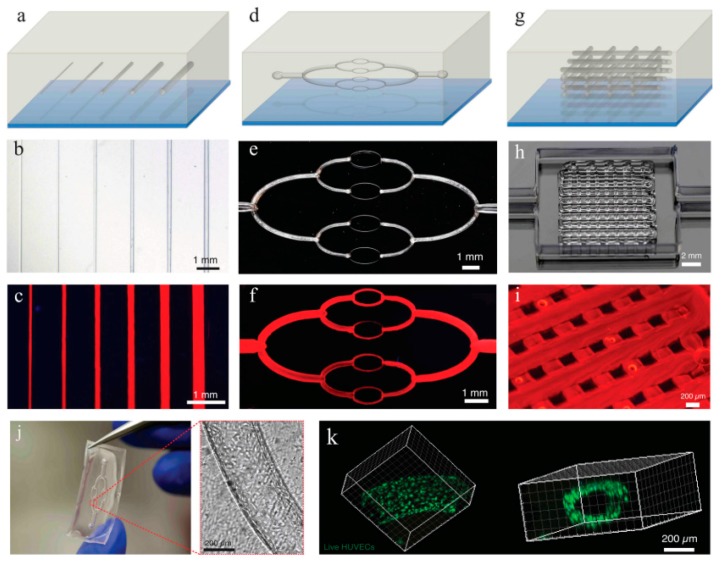
Bioprinted vascular networks perfused with fluorescent dye in: 1D (**a**–**c**); 2D (**d**–**f**); and 3D (**g**–**i**). HUVECS embedded into the 2D vascular network (**j**); and aligned to the walls (**k**). Reprinted from Reference [81], with permission from John Wiley and Sons.

Synthetic bioinks boast high reproducibility and availability. Moreover, they can be chemically modified which makes them versatile and customizable for specific purposes. They possess tunable mechanical properties, but lack adhesion sites for cells, which are the reasons they are frequently blended with natural polymers. Pluronic^®^ is a widely used polyoxyethylene–polyoxypropylene–polyoxyethylene (PEO–PPO–PEO) amphiphilic triblock copolymer (or poloxamer) [80]. It displays thermoreversible gelation behavior above a critical micelle concentration of 20% and a temperature of 10 °C to 4 °C. Due to the thermoreversible character of its gelation process, Pluronic has been used as a fugitive ink for vascular networks [52]. In vascular bioprinting, this sacrificial biomaterial has been employed to support other biomaterials such as fibrin [81] or gelatin [16]. In these studies, the solidified Pluronic is covered with an EC-laden bioink that is then brought to 4 C, when the primary bioink solidifies and the Pluronic liquefies leaving a perfusable channel of ECs (Figure 3). However, the endothelialized channels are able to provide nutrients and oxygen to cells below a certain distance range due to diffusion impediments. Cells further than 1 mm or even less in the case of higher cell density, appear to be in a quiescent state.

Poly(ethylene glycol) (PEG), also known as poly(ethylene oxide) (PEO), is a linear or branched and biologically inert polyether that is widely used in biomedical applications, due to its high hydrophilicity and biocompatibility. PEG is commonly modified using methacrylate (PEG-MA) that, when functionalized at both ends, is commonly termed as PEG-diacrylate (PEG-DA), to enhance its mechanical properties and to permit thermally or photoinitiated polymerization [47]. On the other hand, challenges have been encountered in the bioprinting process [54]. In this work, PEG-DA and tetra-acrylate PEG derivatives (TetraPAc) were co-crosslinked with thiolated hyaluronic acid and gelatin derivatives and compared for their ability to encapsulate NIH 3T3, HepG2, and Int 407 cells. Agarose macrofilaments were used to support the cell-laden bioink. Even if both constructs maintained cell viability up to four weeks, the PEG-DA hydrogels were outperformed by the TetraPAc, not only for the cell response but also for the ease of the bioprinting process. The TetraPAc hydrogels were stiffer which made them retaining the structural integrity during and after bioprinting compared to the brittle and sticky PEG-DA hydrogels. To facilitate the bioprinting process and enhance the biological performance of the materials, PEG modified hydrogels have been used in different bioprinting techniques and have also been combined with other biomaterials, resulting on materials with higher mechanical properties and stabilizing the crosslinked construct [55]. A blend bioink formed by GelMA, sodium alginate, and 4-arm poly(ethylene glycol)-tetra-acrylate (PEGTA) was used to encapsulate HUVECs and hMSCs. In culture, early mature vasculature was observed, which was proved by the expression of CD31 and α-SMA at Days 14 and 21, which indicates early maturation in newly formed vessels.

Within the available synthetic biomaterials for bioprinting, carbohydrate glasses should also be considered [82]. Sugar and sugar alcohol-based glasses are dissolved in water and then boiled to form carbohydrate glasses. These rigid glasses are used as a sacrificial template that will be then surrounded by biocompatible cell laden hydrogels to build perfusable cylindrical channel networks. Carbohydrate glasses have been mixed with dextrans to provide a higher temperature stability and coated with poly(d-lactide-co-glycolide) (PDLGA) to avoid osmotic damage to cells upon extrusion. This strategy allows to create channels with different surrounding biomaterials such as agarose, alginate, PEG, fibrin, and Matrigel.

### 3.3. Material Functionalization

Bioinks alone are not always sufficient to provide the cells with the correct cues for their processes and mechanisms such as proliferation, migration, ECM production and angiogenesis. In Section 2.2, the importance of bioactive molecules in the correct development of vasculature both in the embryo and in the adult human is emphasized. For this reason, growth factors involved in angiogenesis and vasculogenesis have been incorporated in bioprinted constructs. Among the various growth factors used, VEGF is the most commonly used for biomaterial functionalization. VEGF is the main factor responsible of directing vascular development in physiological and pathological angiogenesis [83]. However, high microenvironmental dosage exposure elicits the malformation of vascular networks. VEGF has been incorporated into different bioinks to improve proliferation and migration of cells and final vascular formation. First, this growth factor was incorporated in a fibrin gel printed next to a collagen bioink with encapsulated murine neural stem cells [84]. It was observed that only in the presence of fibrin and VEGF cells change morphology to up to 0.698 mm away from the gel after three days of culture, differentiate and migrate toward the growth factor loaded gel from a 0.5–1 mm distance.

**Figure 4:**
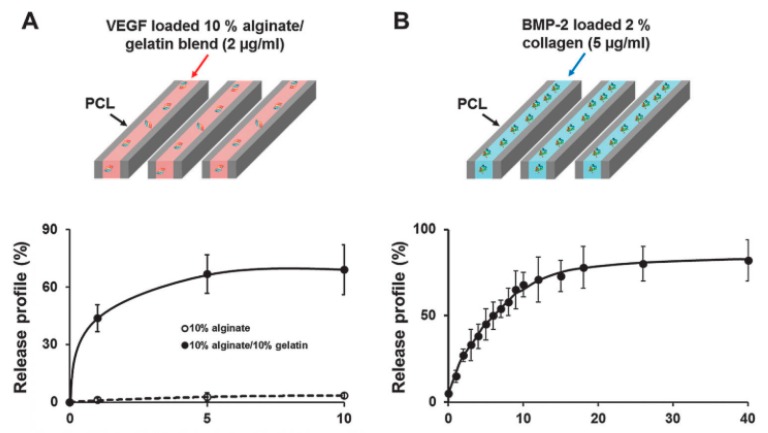
Release profile of: VEGF-loaded alginate/gelatin bioink (**A**); and BMP-2-loaded collagen hydrogel in PCL 3D printed construct (**B**). Reprinted from Reference [46]. Published by The Royal Society of Chemistry.

In another work, gelatin microparticles were bioprinted for the controlled release of VEGF for three weeks [85]. A mixture of Matrigel and alginate was used to encapsulate EPCs in vitro and in vivo, which showed an increase in migration and in the number of vessels in the regions with GMP where VEGF was released. In a later study, a VEGF-loaded alginate/gelatin bioink and a BMP-2-loaded collagen bioink were employed to embed human dental pulp stem cells (DPSCs) in the central zone and in the peripheral zone of a polycaprolactone (PCL) 3D printed scaffold, respectively (Figure 4) [46]. The release of VEGF was slow and reduced by its placement in the central region of the construct which avoid excessive release and consequent vascular abnormalities. In addition, tube-like structures were observed as well as spontaneous vascular formation at the periphery of the scaffold in vivo.

## 4. Cell Sources

Having a good understanding of the in vivo vasculogenesis and angiogenesis processes allows having a deeper view on the different cells and their specific role in the vessel formation and growth processes. Tissue engineering of vascular tissues will therefore be highly influenced by the choice of cell sources and these, at the same time, will be a determining parameter influencing the choice of the biomaterial. In fact, bioprinting gives the possibility to incorporate different cues, additive molecules and factors in the bioink to direct cell fate and/or enhance cell processes such as attachment and spreading.

### 4.1. Cells, Co-Culture Systems and Spheroids

Blood vessels are mainly formed by three layers containing three different cells types: ECs, SMCs and fibroblasts. In the innermost layer, the endothelium, ECs act as a barrier from blood and mediate vascular homeostasis. ECs can be isolated from arteries or veins of different tissues but the most common source is the umbilical cord vein [37,40]. ECs are able to assemble and form microcapillary-like structures without the need for cues or factors [86]. The network structure and the lumen amount depend on the maturation of the network, which can mature in time and be further stabilized when ECs are placed in co-culture with other cells [67].

**Figure 5:**
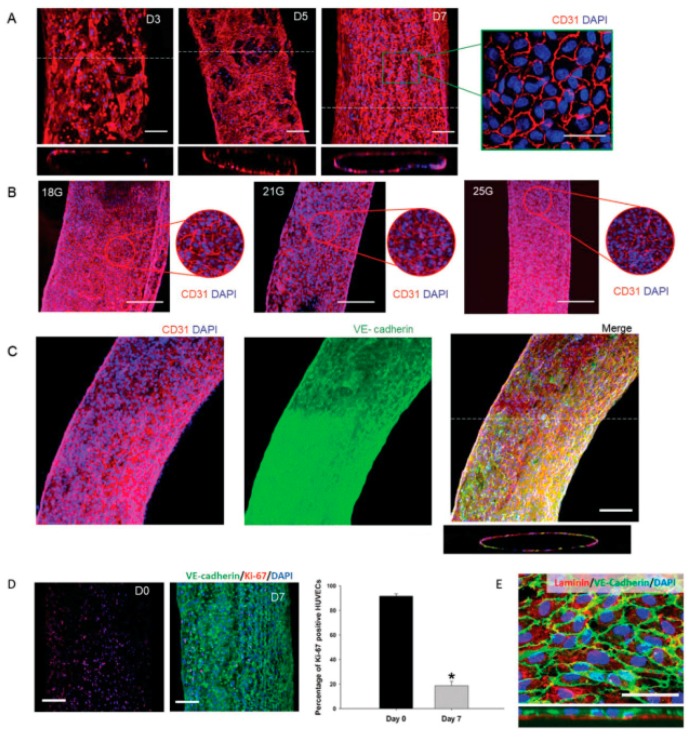
Formation and maturation of the endothelium after three, five and seven days of culture (scale bars are 100 and 50 µm in the inset) (**A**). Vessel formation using different needle diameters for bioprinting (scale bar is 200 µm) (**B**). CD31 and VE-cadherin expression after seven days of culture (scale bar is 100 µm) (**C**). Ki-67 and VE-cadherin staining demonstrated the decrease of proliferation cells and the consequent vessel stabilization (scale bar is 100 µm. * *p* < 0.005, *N* = 3) (**D**). Detection of laminin in the basolateral side of the endothelial layer (scale bar is 20 µm) (**E**). Reprinted from Reference [37], with permission from John Wiley and Sons.

ECs have been used in different bioprinting techniques such as extrusion and inkjet bioprinting [37]. Using extrusion bioprinting, HUVECs were deposited into different flexible vessel-like designs [37]. Immunostaining of CD31 (platelet endothelial cell adhesion molecule) and VE-cadherin (vascular endothelial cadherin) markers allowed visualization of the formation of a confluent vessel-like structure after seven days of culture (Figure 5). After 30 days of culture, the stability of the formed structure was confirmed as a decrease in the number of proliferating cells. Different characteristics of the vessel such as permeability, platelet adhesion, shear stress response and sprouting were also investigated to validate the model.

ECs were also used in inkjet bioprinting, in which hMVECs were bioprinted in fibrin [43]. Cell proliferation following the deposited pattern and tubular structure formation after 21 days of culture were observed.

Other stem cell sources such as (iPSCs)-arterial and venous-like endothelial cells (ECs) [87] and endothelial colony forming cells (ECFCs) [88] have received growing interested in the past years. iPSCs are an attractive autologous source since they are able to give rise to different ECs [87]. However, the subtype and stability of ECs from the differentiation should be controlled and arterial or venous-like ECs should be chosen according to the final target tissue. ECFCs, on the other hand, are circulating cells commonly isolated from cord blood or peripheral blood [88]. These cells are able to mature in ECs and promote angiogenesis. ECFCs are difficult to isolate from humans though. The amount of these cells in the blood is low and even if other sources of ECFCs such as iPSCs have been explored, they are still very complex and difficult to obtain.

Vascular SMCs are located in the intermediate layer of the blood vessels and are in proximity and contact with the ECs in the lumen. These cells have been well studied for their crucial role in vessels structure and function in physiological and pathological conditions [89]. SMCs display high plasticity, a feature required for vessel contraction, cell proliferation and ECM synthesis [4]. However, SMCs are known for their ease to lose their contractive properties as a response to certain environmental factors.

In a recent work, vascular SMCs were bioprinted using a naturally-derived hydrogel as carrier [90]. Aligned microchannels were created to preserve the contractile phenotype of SMCs, which orientate and elongate in the direction of the channels. The expression of α-smooth muscle actin (α-SMA) and smooth muscle-myosin heavy chain (SM-MHC) gene markers was detected even after seven days of culture.

Other cell sources such as iPSCs-derived SMCs [91] or SMCs from endothelial mesenchymal transdifferentiation [92] are very promising sources that have not yet been employed for bioprinting. iPSCs provide an interesting patient-derived source due to their great availability [91]. However, it is still an obstacle to generate protocols that reduce the degree of cell heterogeneity during the differentiation. Furthermore, it was demonstrated that mature bovine vascular endothelium cells can also acquire a SMC phenotype [92]. However, it is still not clear if the transdifferentiated SMCs display plasticity and can maintain contractility.

The third cell component of blood vessels is fibroblasts. This cell type is present in the outer layer of the vessel, the tunica *adventitia*. They are a common cell type from connective tissues and are widely used in vitro. Fibroblasts are derived from MSCs and, when activated, are involved in the synthesis and production of ECM molecules [93]. They also play a determining role during wound healing, contributing to scar tissue formation. In vascular bioprinting, fibroblasts are commonly employed as support cells or in first trials to evaluate cell viability and distribution in new techniques for future vascular channel applications [94]. Gao et al. were able to bioprint L929 mouse fibroblasts in alginate using a co-axial nozzle reaching a viability of 92.9 ± 2.4% after 24 h of culture and even distribution in high-strength structures [94].

Knowing the structure of the native tissue, it is not a surprise that co-culture systems are a valid choice to create a more structured and mature vasculature. A co-culture of HUVECs and fibroblast was employed in a thrombosis-on-a-chip model using 3D bioprinting [95]. HUVECs aligned along the microchannel even in bifurcated channels, which was confirmed by CD31 staining. Co-culture with fibroblasts aimed to more closely resemble the vascular microenvironment in which these cells transform the thrombus into a collagen I-rich clot. High deposition of collagen by fibroblasts was observed in a damaged endothelium model. However, when the HUVECs were forming a continuous microchannel, penetration of fibroblasts and consequent deposition of collagen was difficult to achieve.

Other studies aimed to build more complex vessels through extrusion bioprinting [96] and drop-on-demand bioprinting [48]. Gao et al. bioprinted fibroblasts and SMCs along a rotating rod using a co-axial nozzle while ECs were seeded with collagen inside the hollow tube [96]. After bioprinting, cells were viable and ECs could adhere to the macrochannel. The final construct could be mechanically loaded and thus it could be used in the future for studying angiogenesis and vasculogenesis processes.

Another cell source that has been employed in co-cultures is MSCs, which are multipotent stem cells that are conventionally isolated from the bone marrow and expandable in vitro. MSCs have proved their potential in regulating hemopoietic stem cell function and improving angiogenesis. They can be differentiated into multiple cell types including ECs [97]. Bone marrow-MSCs differentiated into ECs were encapsulated into alginate and bioprinted between two concentric layers of PCL [98]. In this preliminary study, the expression of endothelial markers CD31 and VE-Cadherin was higher in both mRNA and protein levels when pulsatile flow by a peristaltic pump (60 times/minute perfusion rate and 40 mmHg perfusion pressure) was applied during culture. However, a period of four weeks was not enough to achieve cell alignment along the vessel lumen, but it was proved that cell viability after bioprinting was maintained, and it demonstrates the potential of endothelial-like MSCs in vascular applications.

MSCs are also known to be involved in cardiac remodeling and regeneration [99]. HUVECs and MSCs were bioprinted through laser bioprinting on a cardiac patch [100]. Cells were seeded in a vascular-like pattern to favor cell–cell interaction and the co-culture enhanced the HUVECs ability to improve cell survival and vascular differentiation as well as MSCs secretion of factors and ECM. Therefore, angiogenesis and vascular connections were detected in the patch, which was well integrated into the myocardium of RNU rats (strain Crl: NIH-Foxn1rnu) post-infarction. During in vivo transplantation, HUVECs and MSCs were in contact with the damaged myocardium and the cells could form vascular networks as well as improve angiogenesis and remodeling of the left ventricle.

Cell aggregates, namely spheroids, are also another option to engineer vascular construct in bioprinting. Tissue spheroids or microtissues can be self-assembled as building blocks to construct macrotissues or organs [101]. They more closely resemble structurally and functionally the natural tissue environment by improving the intra and extra-cellular communication pathways and therefore have been used in drug screening, matrix remodeling and angiogenesis studies [102]. On a hanging drop culture, embryonic mouse allantoic tissue was cultured and resulted in spheroids with an outer layer of αSMA, SM22-α (smooth muscle protein 22-alpha), and SM-MHC expressing cells and an inner layer of PECAM-1 (also known as CD31), CD34 (hematopoietic stem cell marker) and VE-cadherin expressing cells [103]. Interestingly, only through the presence of endogenous VEGF, fusion of the spheroids and large diameter cavity were observed. However, the hanging drop culture method is time consuming, has a low throughput and it encounters some challenges in controlling the final homogeneity and the size of the spheroid. For these reasons, different homogeneous and heterogeneous spheroids have been developed for vascular applications using different methods [104]. ECs alone are not able to form stable aggregates and disintegrate already after 48 h. On the other hand, ECs in combination with fibroblast or adipose tissue derived mesenchymal stem cells (ADSCs) or a mixture of both, form stable and uniform spheroids in agarose microwells. The addition of ADSCs resulted in spheroid with a larger diameter, a higher ECM production, a uniform HUVECs distribution and a capillary-like network. Furthermore, heterogeneous spheroids constituted by HUVECs and hMSCs were bioprinted through an airflow-assisted 3D bioprinter (technique described in Section 7) [105]. This novel system allowed spiral patterning of cells within an alginate hydrogel. After bioprinting, cell viability was overall 75% and after 10 days, early tubular formation by HUVECs and ossification of osteogenic nodules were detected.

Spheroids of Chinese hamster ovary cells (CHO), or human umbilical vein smooth muscle cells (HUVSMCs), or HSFs, or combinations thereof have been bioprinted for the construction of a scaffold-free tubular structure [35]. Agarose rods and uniform spheroids were fabricated as simple or branched patterns. Spheroids fusion was observed within 5–7 days between HUVSMCs and HSFs. However, to produce larger and longer structures, a more spheroids would be needed resulting in a possible delayed fusion process and non-uniform tubular surface.

### 4.2. Tissue Vascularization

The role of vasculature is to provide tissues and cells with nutrients and gasses, while allowing waste removal. However, vascularization in newly formed tissues remains one of the main challenges in tissue engineering. One of the issues is the slow nature of vasculogenesis and angiogenesis that impede a fast and extended vascularization of the engineered construct. There have been several attempts of introducing and integrating a vascular network within different bioprinted tissues such as the bone [48,49], the liver [41], the cardiac tissue [65], the thyroid [72], the muscle [106] and the kidney [107].

Different 3D bioprinting techniques have been combined to build a vascularized bone structure [108]. To mimic the hierarchical structure of bone, a biphasic scaffold mimicking a hard mineral core surrounded by soft matrix was created combining fused deposition modeling (FDM) and stereolithography (SLA). The central part mimicking the mineral core of bone was fabricated with biodegradable polylactic acid (PLA) using FDM printing. PLA fibers were deposited creating an interconnected vascular network formed by a central channel (≈1.5 mm) surrounded by small ones (≈200 µm) and further functionalized with BMP-2 peptide using Michael addition reaction onto a polydopamine coating. After manufacturing, hMSCs were seeded on the PLA. Then, using SLA, hMSCs and HUVECs were bioprinted together in a 10 wt% GelMA bioink functionalized with VEGF peptide via click chemistry. Cells were deposited into the PLA channels to mimic bone vascular network. BMP-2 functionalized PLA showed that higher hMSCs proliferation was achieved after five days of culture. However, in the GelMA layer, the cell viability initially dropped due to UV curing process, but it increased over the culture period. During dynamic culture, the channels were perfused using a peristaltic pump with a 5 mL/min^−1^ flow to avoid high shear stresses. Perfusion was necessary to achieve a primitive vascular network since in static culture, cells in the hydrogel tend to assume a round morphology. HUVECs formed primitive tubular structures after seven days of culture. Less capillary-like networks were present in the controls without VEGF. After four weeks of culture, osteopontin (OPN), von Willebrand factor (vWF) and CD31 stainings were performed. OPN and vWF stainings showed that constructs with VEGF and BMP-2 enhanced osteogenesis and angiogenesis while CD31 showed the formation of the capillary network. Further confirmation of successful osteogenesis and angiogenesis processes in BMP-2 and VEGF functionalized constructs was performed by measuring alkaline phosphatase (ALP) activity, collagen type I (Col I) expression, VEGF secretion, and calcium deposition.

In a bioprinted construct for liver tissue engineering, angiogenesis was achieved through co-culture of parenchymal and nonparenchymal stem cells [41]. In a collagen bioink, hepatocytes (HCs), HUVECs and human lung fibroblasts (HLFs) were co-cultured into a PCL framework. This co-culture system had the aim to provide a 3D culture environment to enhance viability and functionality of HCs by interaction with other cell types and with the ECM. Functionality of HCs was evaluated through urea synthesis, an indicator of HCs metabolism and albumin secretion, a protein synthetized by HCs. HLFs are known to contribute to angiogenesis releasing soluble growth factors for ECs. The formation of a capillary network was achieved with a mixture 1:3 of HUVECs and HLFs. After 14 days of culture, HUVECs sprout and formed capillary-like tubes with lumen. In addition, survival and functionality of HCs was enhanced by the presence of HUVECs and HLFs. Increased viability, albumin secretion and urea synthesis during 10 days of culture demonstrated that the co-culture system is a better option compared to single cells culture.

**Figure 6:**
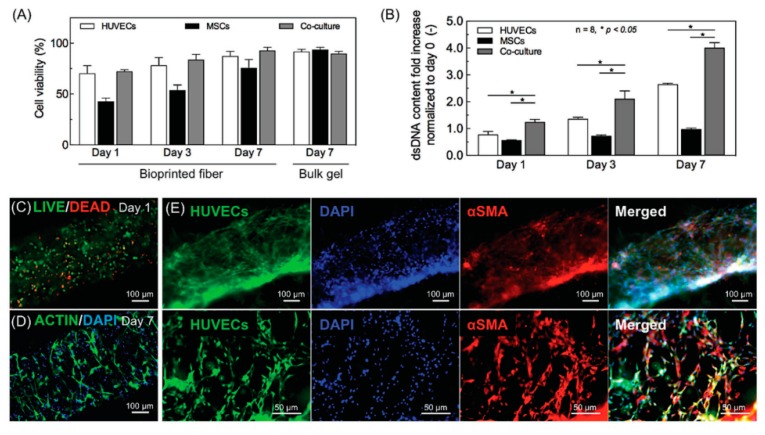
Cell viability and cell proliferation of HUVECs, MSCs and co-culture after one, three, and seven days of culture (*N* = 8; * *p* < 0.05) (**A**,**B**). Live/Dead staining at Day 1 for cell viability (**C**) and F-Actin/DAPI after seven days of culture for cell spreading (**D**). Co-culture of GFP-HUVECs and MSCs in the bioprinted capillaries after seven days of culture. Differentiation of MSCs into perivascular cells was confirmed by staining of α-SMA. Reprinted from Reference [48], with permission from John Wiley and Sons.

In another example, GelMA functionalized with VEGF was used as bioink to fabricate a bone vascularized tissue construct (Figure 6) [48]. To obtain two different niches, one for vascularization and one for osteogenesis, the final structure was designed with two different types of GelMA. First, a central fiber was bioprinted using 5% VEGF-loaded GelMA with HUVECs and hMSCs. Then, around the fiber, three layers of GelMA rods loaded with silicate nanoplatelets, VEGF and hMSCs were bioprinted to mimic the bone niche. The nanoplatelets were used to induce osteogenic differentiation of hMSCs while gradient concentrations of VEGF for vascular spreading. In the central rod, the fast hydrogel degradation allowed the alignment of HUVECs and hMSCs along the channel walls. Furthermore, hMSCs differentiated into SMCs, which promoted not only the proliferation of HUVECs but also angiogenesis and consequent stabilization and maturation of the capillary-like structure. During the long-term culture, the bone niche was formed and survived thanks to the central core vessel.

**Figure 7:**
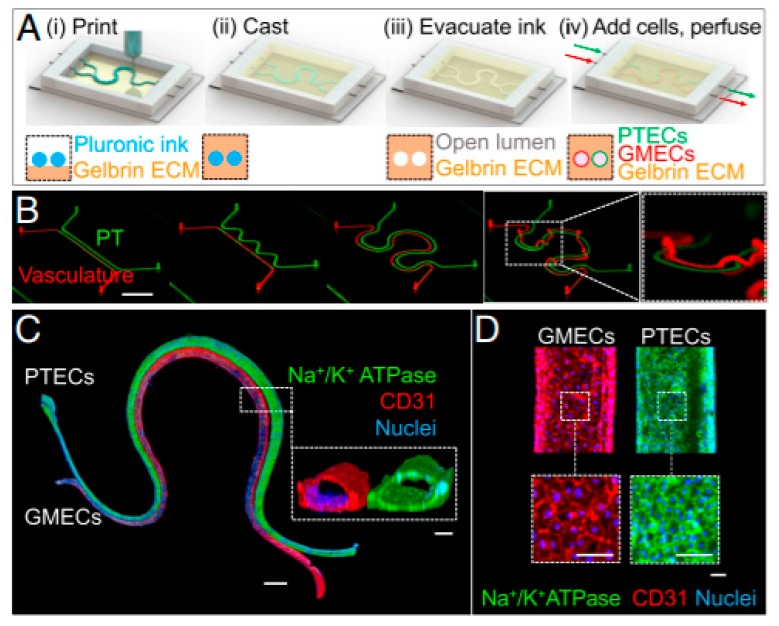
Schematic illustration of the fabrication of a vascularized 3D bioprinted model (**A**). Different design patterns ranging from straight to serpentine (Scale bar is 10 mm) (**B**). Immunostaining of 3D final VasPT model (Scale bar is 1 mm) and cross-section (inset) of the PT and vascular conduits (scale bars are 100 μm) (**C**). High magnification of the two lumens (scale bars are 100 μm) (**D**). From Reference [107], with permission from Proceedings of the National Academy of Sciences of the United States of America (PNAS).

In a recent work, bioprinting was employed to fabricate tissue engineered kidney and to study the selective renal reabsorption of albumin and glucose in the tissue (Figure 7) [107]. A 3D vascularized proximal tubule (3D VasPT) chip was fabricated using Pluronic as fugitive ink deposited in modified ECM, mainly made of fibrin and gelatin. Pluronic was mixed with high-molecular-weight poly(ethylene oxide) to retain the bioprinted pattern after ECM deposition and removed by cooling and liquefying. After Pluronic removal, the two channels were perfused with proximal tube epithelial cells (PTECs) and glomerular microvasculature endothelial cells (GMECs). The final construct had colocalized proximal tubule and vascular channel. PTECs and GMECs reached confluency in the chip in the first 3–5 days of culture. To prove that the two cell types reached maturity, they were stained with different markers. PTECs were marked with Na+/K+ ATPase, sodium-glucose cotransporter-2 (SGLT2) and basement membrane protein (laminin). Primary cilia were also observed on the surface of PTECs. As the channels started to maturate, the number of cells decreased reaching stability. GMECs were stained for endothelial marker CD31 and vWF and the formation of adherent junctions was confirmed and visualized by transmission electron microscopy (TEM). To study renal reabsorption, renal and vascular channels were separately perfused by a closed-loop system and albumin and glucose reabsorption was measured as indicators of renal correct function. When the proximal tubule was perfused with Cy5-conjugated human serum albumin (HAS-Cy5), it was found that the albumin uptake increased after 1 h and the presence of HAS-Cy5 around the proximal tubule and the transport between lumens was confirmed on the vasculature. Furthermore, glucose reabsorption was investigated by using a glucose meter. Glucose levels increased during culture time, starting from Day 1 in which PTECs reached confluency until reaching a plateau on Day 8. The reabsorption was 5–10-fold higher in the 3D model chip compared to 2D transwell controls with pre-coated ECM membranes. In conclusion, it was proved that the 3D VasPT is a valid model to study renal reabsorption in which mature and confluent epithelium of PTECs and endothelium of GMECs can be perfused with different soluble and drugs to investigate the epithelium-endothelium interaction.

More recently, a prevascularized muscle construct was generated using a granule-based printing reservoir for the treatment of volumetric muscle loss (VML) [106]. A co-axial nozzle was used to deposit multiple bioinks into a gelatin bath. The bioinks were obtained from decellularized ECM (dECM) of skeletal muscle and vascular tissue of porcine tibialis anterior muscles and descending aortas, respectively. During bioprinting, the dECM gels quickly coagulated inside a bath containing gelatin granules and polyvinyl alcohol (PVA). The addition of PVA as a co-agent improved the stiffness and stability of the final construct by increasing the triple helix structure of dECM collagen. Muscle dECM (mdECM) and vascular dECM (vdECM) were used to encapsulate human skeletal muscle cells (hSKMs) and HUVECs, respectively. Compared to other biomaterials such as collagen, fibrin and GelMA the vdECM bioink exhibited higher ECs proliferation and gene expression after 1, 4, 7 and 14 days of culture. Three different constructs with 200 μm channels were created: a printed structure with only hSKMs in mdECM, a mixed structure with hSKMs and HUVECs together in mdECM and vdECM and a co-axial one with an inner core of hSKMs in mdECM and an outer shell of HUVECs in vdECM. Myotube and capillary-like network formation were detected by muscle myosin heavy chain (MHC) and CD31 staining respectively. Throughout the co-axial printed structure, fully developed myotubes and ECs networks were present in the core and in the outer part, respectively. On the other hand, in the mixed printed construct, MHC and CD31 revealed few and partially formed myotubes and ECs network that could not co-exist in the same area. It was hypothesized that during co-culture the two cell types interfere in each other proliferation and differentiation processes. Furthermore, a contractile force measurement showed that among the three constructs, the co-axial printed one has the highest twitch and tetanic peak forces, which demonstrates the functionality of the final fabricate. In an in vivo rat tibialis anterior VML injury model, the prevascularized co-axial printing construct showed higher muscle weight and functional recovery, higher muscle fiber and blood vessel formation and less fibrotic tissue compared to the other two tissues. Considering the results of the in vivo model, this construct was also used to investigate neuronal integration. Neuromuscular junction staining by Alexa594 conjugated α-bungarotoxin confirmed the integration with the in situ neural network. However, it is still a challenge to create large vascularized constructs, which are often required in extensive VML defects. Large constructs exhibit hypoxia regions and limited nutrients and oxygen diffusion, which lead to cell death.

Previously cited works have mainly focused on creating capillary-like structures, taking advantage of HUVECs self-assembly properties. In this way, nutrient and oxygen diffusion can be achieved even when multiple cell types are present. However, these works have shown limitations in bioprinting clinically-relevant cells (mainly cell lines have been used so far) and in elaborating vascularized constructs. To more closely resemble tissue formation and function, a branched vascular network able to undergo angiogenesis, remodeling and regression with relevant cell sources would be required.

## 5. Bioprinting Techniques

Bioprinting is an additive manufacturing process in which cells, biomaterials and bioactive molecules (soluble molecules that interact and modulate the cell activity, such as growth factors, cytokines or hormones [109]) are combined and assembled to fabricate bio-engineered structures for biological study and particularly for regenerative medicine [110,111]. The combination of these compounds is known as bioink [112]. In comparison with conventional additive manufacturing using cell-free scaffolds, bioprinting includes living cells during the process of deposition, which requires special measures due to their sensitivity to temperature and mechanical tension as high pressure and shear stress, as well as pH and biocompatibility of surrounding materials. The 3D construct to be deposited is first designed using CAD software to obtain a 3D model. This model is then sliced into layers and each layer is decomposed in a succession of patterns to fill the full construct. Finally, the ready-to-deposit design is converted into a machine code (e.g., G-code, often used as a universal code for printers), which is read by the printer as a succession of instructions including various parameters such as pressure, feed rate and coordinates.

To date, in the field of bioprinting, there are four main strategies that fit with the previous mentioned requirements: extrusion-based, drop-based, laser-based and stereolithography bioprinting [113]. Each technique has its own requirements and characteristics translated into specific advantages and limitations. Moreover, each technique accounts for a different range of resolution, manufacturing time and design limitation [110,114]. In addition to the intrinsic technique properties, bioprinting can be carried out as the fabrication of one or multiple materials simultaneously.

To produce vascular channels using extrusion-based bioprinting (Bioplotting), most of the literature refers to cell-free inks deposited as a sacrificial hydrogel embedded within an ECM-like hydrogel in order to produce, after removal, a hollow tubular construct that is later seeded with cells [16,40,81,115]. Mainly, the cells are loaded in a gel that is manually deposited surrounding the sacrificial ink. Here, we consider only a bioink, and thus bioprinting, the technique that involves the deposition of a cell-laden material, others related to cell-free material are considered as regular ink.

### 5.1. Extrusion-Based Bioprinting

The most common technique, also named Bioplotting, consists on dispensing a continuous filament of cell-laden bioink through a nozzle using a piston, a screw or pneumatic system to build a 3D construct layer-by-layer. This technique provides an accurate bioink deposition (resolution: >100 µm) and a homogenous cell distribution with relative great speed of processing. In addition, the bioinks can reach a very high cell density and an excellent structural integrity due to the continuous deposition of the bioink [116]. A large range from low to high viscosity biomaterials, from 30 mPa/s to >6 × 10^7^ mPa/s [18,117,118], can be extruded through a range of nozzle sizes. Nevertheless, for bioprinting applications, many significant limitations remain. It is important to consider that higher viscosity bioinks lead to increased shear stress and decreased cell viability as well as to a limitation on lower nozzle sizes. According to Smith et al., considering the comparison between elongated Bovine aortic endothelial cells (BAECs) and round BAECs incubated 24 h after bioprinting, they observed 46% of viability using 33 G nozzle (0.108 mm of inner diameter) against 86% with a 25 G nozzle (0.260 mm of inner diameter) [119]. Indeed, the pressure due to the extruding process can induce a distortion or breach of cellular membranes that affect cell viability, with extreme extrusion pressure the cell viability can drop down to 40% [120,121]. In addition, to improve the cell viability, biomaterials should resemble as much as possible the native ECM. That is, hydrogels need to possess a specific range of stiffness and biodegradability in order to let the cells spread through, which can restrain the choice of biomaterials [17,122].

**Figure 8:**
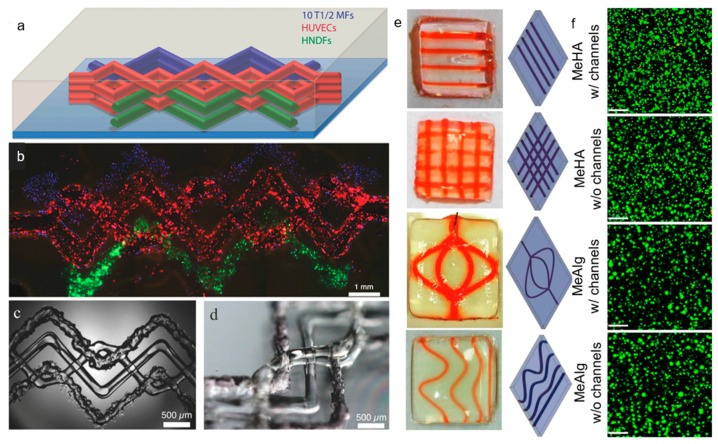
Vascular channels using extrusion-based bioprinting. (**a**) Schematic view of a heterogeneous engineered tissue construct, in which blue, red and green correspond to bioprinted 10T1/2 fibroblast-laden GelMA, fugitive ink Pluronic F127, and GFP HNDF-laden GelMA, respectively. Note that red filaments are flushed out to form hollow channels seeded afterwards with RFP HUVECs. (**b**) Composite image of the 3D bioprinted tissue construct using fluorescence. (**c**) Bright field microscopy image of the 3D bioprinted construct. (**d**) Image showing the spanning and out-of-plane nature of the 3D bioprinted construct. (**a**–**d**) Reprinted from 3D Bioprinting of Vascularized, Heterogeneous Cell-Laden Tissue Constructs, Kolesky et al. Copyright (2014), with permissions from Wiley [81]. Showing the extrusion of different bioinks into an acellular ECM in order to produce vascular channels. (**e**) 3D bioprinted devices and corresponding digital designs. (**f**) Confocal scanning microscopy images of hMSCs Live/Dead assay, bioprinted with MeHA and MeAlg bioinks with and without channels and cultured for one day (scale bar: 200 µm). (**e**,**f**) Reprinted from 3D bioprinting of complex channels within cell-laden hydrogels, Ji et al. Copyright (2019), with permission from Elsevier [53] showing the extrusion of sacrificial ink (perfused with a red dye afterward) into a bioprinted bioink in order to produce a vascular channel.

As mentioned above, there are several systems related to extrusion bioprinting, and each of them has pros and cons. Pneumatic systems are usually associated to a delay in dispensing due to the compressed gas, but can support highly viscous biomaterials. Piston-driven deposition provides a better control over the flow, while the biomaterial extrusion in screw-based systems offer a better spatial control [123]. To fabricate vascular structures, a novel strategy arose in the past decade. The templating technique or sacrificial templating is a convenient and relevant approach to produce tubular structures using extrusion-based bioprinting. This technique allows the use of a wide range of leachable materials (Pluronic F127 [16,81,107,124], carbohydrate glasses [82], gelatin [71], agarose [47], alginate [125], PVA [126] and poly(*N*-isopropylacrylamide) (PNIPAM) [127]) and consists in embedding a matrix hydrogel around a leachable template that can be removed once the matrix is jellified. Removal of the fugitive ink results in the formation of an empty structure. As an example of a cell-laden bioink approach to the formation of vasculature, Ji et al. deposited a sacrificial ink between several layers of a cell-laden bioprinted hydrogel bioink in order to produce a perfusable channel into a cellularized matrix (Figure 8e,f) [53]. They deposited a hydrogel matrix composed of either methacrylate alginate (MeAlg) or methacrylate hyaluronic acid (MeHA) as a support hydrogel, and partially cured it for few seconds (15 s for MeAlg and 5 s for MeHA) between each deposited layer. Then, they deposited the sacrificial ink (Pluronic F127, 40%) on top of the matrix hydrogel and finally covered it again with several layers of matrix hydrogel following the same process exposed above. Once the construct was done, they photopolymerized the full construct for several minutes (4 min for MeAlg or 1.5 min for MeHA). After the final curing, the construct was immersed in PBS to dissolve the sacrificial ink and obtain a perfusable channel. hMSCs were included in the bioprinted matrix hydrogel and HUVECs were perfused inside the channel after injection of fibronectin to promote the cell attachment. Adjusting the feed rate and the pressure, they could produce fibers from 85 µm to 1.2 mm with a 34 G needle (0.0826 mm of inner diameter) and 30 G needle (0.159 mm of inner diameter), respectively, but after perfusion the channels appeared to be bigger than 250 µm. Their approach allowed producing channels with a large range of sizes and complexities [53]. In this study, Ji et al. developed a novel approach to sequentially bioprint and crosslink layers of a cell-laden hydrogel that allowed to support the sacrificial ink during deposition. It is also important to notice that the sacrificial ink and the matrix hydrogel did not mix during the deposition, allowing a good resolution of the final channel after perfusion. Despite the possibility to adjust the deposition of the fugitive ink and produce a multistage channel by sequentially crosslinking the matrix, this technique does not allow a fully free-form deposition and the necessity to keep the construct in a humid environment to avoid shrinking is a significant limitation.

In a similar approach, Kolesky et al. bioprinted a cell-laden hydrogel, GelMA containing human neonatal dermal fibroblasts (HNDFs), within an acellular ECM-derived hydrogel, pure GelMA, enabling the fabrication of a custom cell patterning surrounding a perfusable channel seeded with HUVECs. Here the channels were produced using different thermal transitions between the sacrificial ink (Pluronic F127), which is liquid below 4 °C, and the GelMA which is, conversely, solid below 23 °C. GelMA was bioprinted at 37 °C and then cooled down to induce gelation; the sacrificial ink was deposited on top, prior to being covered with a second layer of GelMA. Then, the construct was cooled down to 4 °C to remove the Pluronic and obtain a hollow tubular channel [81]. In this case, the sacrificial ink does not contain cells as the matrix hydrogel while the GelMA fibers printed around the vascular channel does. In the same study, Kolesky et al. increased the complexity of the model by combining different cell types, HSFs, mouse fibroblasts (10T1/2) and HUVECs, achieving different cell viability depending on the cell type, primary and cell lines of human and animal origin in the ECM. They assessed a cell viability of 61% and 70%, for the mouse cell line and the primary HDFs, respectively, at Day 0 with an increase to 82% and 81% at Day 7. In this example, Kolesky et al. used a similar approach including cell-laden GelMA in combination with the sacrificial ink (Figure 8a–d) [128]. Compared to the study performed by Ji et al., their study shows the possibility to bioprint together two cell-laden fibers and print a perfusable channel within an acellular matrix. The innovative approach in this study is the combination of different cell types in a single thick tissue including a vascular channel. Since a physiological tissue is composed by several cell types working in synergy, being able to produce a single tissue including a vasculature allowing the interaction between several embedded cell types make this model relevant to study the cell interaction in a complex environment.

### 5.2. Drop-Based Bioprinting

Drop-based bioprinting, also named inkjet or drop-on-demand technique, is based on the ejection of a cell-laden bioink out of a nozzle head in the form of droplets via thermal, piezoelectric or electrostatic actuators to selectively allow the drops to fall on a substrate with a precise control over the position using a non-contact approach [129]. Adjusting different parameters, such as the valve aperture time, feed rate and pressure applied, make it possible to regulate the volume of droplet and the cell density, and thus the average number of cells per drop. This technique provides a higher resolution than extrusion-based bioprinting (resolution: 20–100 µm) but with a lower manufacturing time in terms of driven volume per minute. Drop-based technique allows depositing living cells in liquid solution or low viscosity hydrogel. Nonetheless, because of the low viscosity of the bioink, the depositing process has to be relatively short, otherwise cells would aggregate in the suspension that can disturb the drop formation and the trajectory during deposition and, consequently, reduce the bioprinted resolution as well as the homogeneous cell distribution in the final construct.

**Figure 9:**
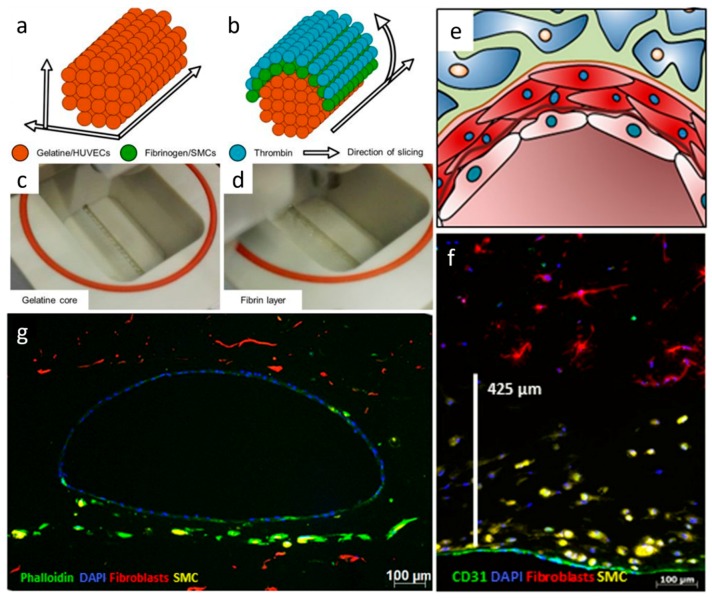
Printing procedure to manufacture vascular channels in custom-made bioreactors. (**a**) The gelatine core is deposited by following a printing pattern, which is created by slicing the channels horizontally and calculating the droplets in a grid for each longitudinal section. (**b**) For the surrounding fibrin layer, the deposition pattern is calculated by slicing the channel lengthwise and defining droplet positions in angular steps around the channel outline of each cross section. (**c**) To deposit the gelatin core, a single printer head is used at 37 °C. (**d**) Two printer heads are used, filled with fibrinogen and SMCs alternatingly, as well as thrombin as a crosslinker. (**e**) Schematic cross section of the channel in close-up shows a single layer of ECs and an additional SMC layer. (**f**) Combination of SMC layer and the EC layer showing the distribution of SMCs close to the intact channel as a cross section after seven days of flow (CD31 in green, pre-labeled fibroblasts in red, pre-labeled SMCs in yellow, DAPI in blue, 20×). (**g**) A representative fluorescence microscopy image in cross-section highlights the whole architecture of a multi-layered vessel model after four days of dynamic cultivation; EC are homogenously distributed in the inner part of the lumen, surrounded by fibroblasts and SMCs (phalloidin in green, pre-labeled fibroblasts in red, pre-labeled SMCs in yellow, DAPI in blue, 5×). (**a**–**g**) Figure adapted from Schöneberg et al., 2018. Engineering biofunctional in vitro vessel models using a multilayer bioprinting technique. Scientific Reports, Copyright (2018) [21].

Several strategies have been investigated to produce vascular constructs using drop-based bioprinting. As briefly mentioned in Section 3.1, Schöneberg et al. used drop-based bioprinting to generate in vitro blood vessel models mimicking the physiological composition of a native vascular channel [21]. Their approach was to deposit droplets of three different cell types embedded in different hydrogels in a defined spatial arrangement to mimic the different layers of a natural vessel. Three bioinks distributed in three different printheads were used: the first one containing 3 wt% gelatin with HUVECs (10–12 million/mL) at 37 °C; the second one containing the crosslinker solution (thrombin); and the third one containing 2.5 wt% fibrinogen with SMCs (6 million/mL). The bioreactor in which the bioinks were deposited was kept at 5 °C during all the bioprinting process. To produce the perfusable channel, they deposited the first bioink as a succession of droplets to form a removable (sacrificial) rod. Then, the second bioink containing the crosslinker was deposited directly followed by the deposition of one drop of the third bioink containing the SMCs and fibrinogen. The last two steps were repeated until the entire sacrificial rod was covered. This approach allowed the crosslinking of the fibrinogen in fibrin as a thin layer around the core material (Figure 9a–d). Once the process was completed, the reactor was filled with a mixture of collagen and fibrinogen containing fibroblast cells, which was quickly crosslinked with thrombin 8 U/mL. Finally, they gently flushed out the core material with PBS at 37 °C to obtain the open channel [21]. This approach is interesting as they succeeded to reproduce the three layers of a blood vessel by reconstituting the *tunica intima* (endothelium), *tunica media* (elastic SMCs) and the *tunica adventitia* (matrix of fibroblast) (Figure 1 and Figure 9e–g). Moreover, unlike most of the approaches related to sacrificial templating, this strategy leads to the endothelialization of the inner part of the channel without a post-seeding step and shows the formation of a confluent and continuous endothelium inside the lumen after four days in culture.

Nishiyama et al. investigated a different approach to fabricate a tubular structure using drop-based bioprinting technology. Droplets of alginate were deposited at the surface of a CaCl_2_ bath that induced the crosslinking of the hydrogel and produced beads on the surface of the substrate bath. By repeating the same pattern at the same location, the construct was slightly drowned, allowing the next layer to be in contact with the substrate solution. To maintain the shape of the construct through several layers, they had to improve the viscosity of the alginate beads, by addition of a viscosity enhancer. It appears that PVA was the best candidates among dextran, hyaluronan, xanthan gum, guar, carrageenan, tragacanth gum, and pectin, in terms of cell biocompatibility, homogeneous dispersability and stability. To fabricate a tubular structure, they deposited a round pattern of alginate beads, which were merged to form rings. By superposition of multiple stacks, the rings formed a tubular construct. They reproduced the same structure including HeLa cells (6 million/mL) in the 1 wt% sodium alginate and immerging in a 10 wt% calcium CaCl_2_ (with PVA, 15 wt%) and succeeded to obtain a cell-laden tubular structure [38]. This last approach has the advantages of being fast, relatively simple and allowing the production of long tubular structures containing cells. Nevertheless, this technique does not offer the possibility to fabricate intricate structures or bifurcation vascular models and a more representative model than HeLa cells would have been more relevant to vasculature. To summarize, drop-based bioprinting is a powerful technique to produce tubular structures mimicking the physiological composition but remains underexploited in the field of vascular bioprinting.

### 5.3. Laser Assisted Bioprinting

Laser Assisted Bioprinting (LAB) or Laser-Induced Forward Transfer (LIFT) utilizes a laser pulse to detach cells from a donor slide, depositing them on a given substrate following a specific pattern [110]. Most commonly, the donor slide, or ribbon, is a metal slide under which a cell layer is deposited as homogeneously as possible. Upon receiving the laser pulse from the top side of the donor slide, the metal absorbs the energy that induces the formation of a bubble underneath the cell layer and leads to the cell detachment [20]. This technique combines the highest cell viability and highest resolution (unique cell deposition) from the three previous mentioned techniques. Indeed, adjusting parameters such as the bioink viscosity and thickness, laser energy, surface tension, wettability, and the air gap between the substrate and the donor slide, it is possible to accurately control the droplet volume (from 10 to 7000 pL). Moreover, LIFT allows depositing high cell densities of up to 10^8^ cells/mL and high viscosity materials (from 1 to 300 mPa·s) [108]. Nevertheless, this technique is also one of the least productive in terms of volume bioprinted per minute and preparation of samples [110]. Indeed, cell suspensions need to be loaded on the bottom of the donor slide and to stay humid during all the bioprinting process that require an extremely high relative humidity percentage in the bioprinter enclosure to counter effect cell dehydration [130,131,132]. Wu et al. exposed that, even with a relative humidity near 100%, the bioprinting process encounters troubles for the deposition over a period of time as short as 10 min, partially due to the movement of the donor slide during processing [68,132]. In addition, having a homogeneous distribution of the cells on the donor slide remains a challenge regarding the drying effect and the gravity induced modification of the cell layer, leading to the formation of cell aggregates. To counter balance the evaporation of the droplet or cell suspension on the donor slide, it is possible to add a supplement as 10 wt% glycerol in sodium alginate solution that can increase the time allocated to deposit the droplets [131,133].

**Figure 10:**
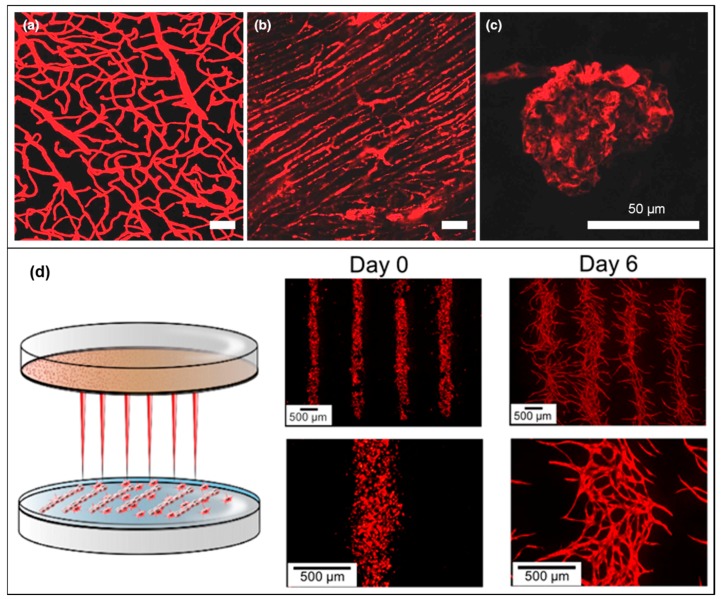
Examples of tissue dependent micro-vascular patterns. (**a**) Micro-vessels of a mouse brain following a space-filling morphology. (**b**) Micro-vessels of a mouse heart adjusting their morphology to align with cardiomyocytes. (**c**) Micro-vessels of mouse kidney organized as glomeruli (all scale bars are 50 µm). Reprinted from Understanding vascular development, Udan et al. Copyright (2012), with permissions from Wiley [134]. (**d**) Laser-assisted bioprinting (LAB) technology (left) to optimize the bioprinting of HUVEC lines (middle) and their maturation into a pre-oriented micro-vascular network after six days of culture (right). Reprinted by permission from Copyright Clearance Center: Springer Nature, Journal of Materials Science: Materials in Medicine, Micropatterning of endothelial cells to create a capillary-like network with defined architecture by laser-assisted bioprinting, Olivia Kérourédan, Jean-Michel Bourget, Murielle Rémy et al., 2019 [42].

Vascular patterns can differ from one tissue to another (Figure 10a). Vascular networks in the brain aim to fill the space (Figure 10a), while vascular networks located on the heart are aligned with cardiomyocytes (Figure 10b) and for the kidney, the vessels form glomeruli (Figure 10c) [134]. Hence, it seems important to reproduce a physiological vascular network with a relevant pattern for a specific localization. The major asset of LIFT, is the control and the resolution of the drop deposition. This particularity offers the possibility of the deposition of pre-oriented arrays of cells in order to induce the growth and maturation of cells following a specific pattern. Wu et al. investigated if a specific pattern can influence the orientation of the cell growth, the lumen size or the shape of the vascular network. They used a biological laser printer (BioLP) to produce pre-oriented lines of ECs (i.e., HUVECs and HUVSMCs) following a leaf vascular custom patterning. Bioassembly is a biofabrication technique consisting on the fabrication of a hierarchical construct through the automated assembly and reorganization of a material or cell, named also self-assembly. ECs can self-assemble and maturate in a vascular-like network in presence of growth factors. Wu et al. took advantage of this particularity to guide cell proliferation and maturation to produce a vascular-like network. Since a vein vasculature has similarities with a leaf vasculature, they designed a branched structure based on the leaf vasculature to provide a framework for the cells to grow and from a network with 50–150 µm of space between cell droplets. The deposition of HUVECs following the mentioned pattern allowed the formation of an interconnected network. After 20 h of culture post-deposition, HUVECs were already connected to each other. A comparison between the interconnection of HUVECs cells in function of the droplet-to-droplet distance showed that cells formed a wider structure with 50 µm of spacing than with 100 or 150 µm droplet spacing. This early connection between cells was due to the substrate as they used Matrigel containing chemoattractant and VEGF. As a result, the cell density, distance from each other and orientation influenced the proliferation and maturation of cells into a vascular structure. In addition, LAB remains a strong technique to study cell–cell interactions in terms of spatial organization [132]. Kérourédan et al. also studied the micro-patterning of ECs to obtain a custom micro-vascular network using LAB (Figure 10d). The innovative approach was to combine endothelial progenitor cells (EPCs) with stem cells from the apical papilla (SCAPs) as osteoprogenitors, to enhance bone formation due to the formation of a vascular network. In their study, they deposited a specific pattern of EPCs on a collagen hydrogel previously seeded with SCAPs aiming at promoting vascularization around and inside the bone substitute. The cell droplets were deposited as lines in a first approach, and then deposited to form circles. The formation of a capillary-like network that grew following the induced pattern was visible at Day 6 (line) and Day 3 (circles) (Figure 9d) [42]. Previous works showed the capability to print cells in situ using LAB bioprinting [135,136]. Combining the previous mentioned studies, they investigated the deposition of ECs in situ in a bone calvaria defect to promote bone regeneration through pre-vascularization. The critical size bone calvaria defects were prefilled with a MSCs loaded collagen hydrogel prior the deposition of specific HUVECs pattern. They observed, after two months in vivo, a vascularization and bone regeneration rate (respectively, vr and br) significantly higher with “disc” (vr = +203.6%; br = +294.1%) and “crossed circle” (vr = +355%; br = +602.1%) patterns than a “random seeding” (reference) [137]. This study shows the impact of patterning on the vascularization and bone regeneration but also the possibility of accurately deposit cells in situ for in vivo applications.

Instead of pre-orienting the cells to control over the specific endothelial growth and maturation patterning, Gruene et al. studied the cell–cell and cell–environment interaction through multicellular micro-patterning using LAB. The purpose was to investigate, as a proof of concept, the vascular-like network formation through the cell interactions. Here, adipose-derived stem cells (ASCs) and human endothelial colony-forming cells (ECFCs) were deposited onto a natural hydrogel composed with fibrin and hyaluronic acid with a layer-by-layer approach using a LAB. A first layer of hydrogel was produced on a collector slide and crosslinked prior to deposit on top, the different cell types controlling the space between each drop and the amount of cells per drop. They repeated this step several time to obtain a 3D environment. Then, they evaluated the cell behavior, adjusting the cell amounts, cell ratio and the distance between cell droplets. The interesting approach in this study is related to micro-patterning and the control over the distance between cells (ECFCs deposited as a 9 × 9 spot array with 800 µm spot spacing; ASCs deposited as an 8 × 8 spot array with a 400 µm spot spacing) in a 3D environment that is possible using the LAB. Moreover, the viability of the cells have been evaluated compared to a non-bioprinted control, showing that neither cell type is affected by the LAB procedure regarding viability, proliferation and DNA fragmentation (99.7% ± 0.6% for ASCs and 97.8% ± 3.1% for ECFCs compared to the non-bioprinted cells). As a result of this study, they could establish the effect of a direct contact between the ASCs and ECFCs on the formation of a vascular-like network, as the network formation during Days 3–5 in the case of co-culture corresponds to the only time point where the cells are in contact [44].

To summarize, LAB can be used as a model to guide the proliferation and maturation of ECs inducing a self-assembly process that leads to the formation of a pattern-specific vascular-like network. LAB could also be exploited to study cell–cell interactions in a controlled environment.

### 5.4. Stereolithography Bioprinting

**Figure 11:**
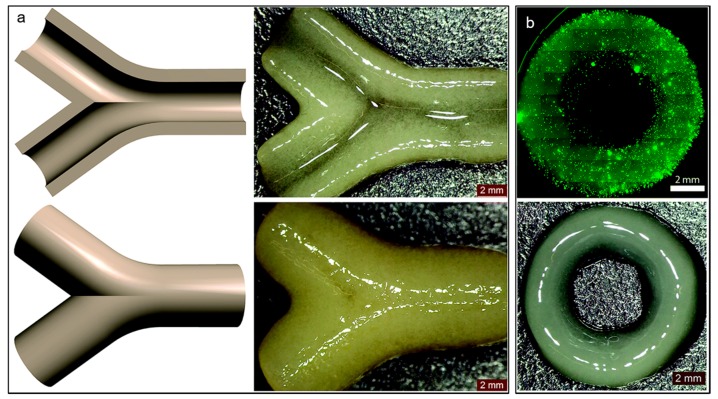
Bifurcation vascular tubes and cell-laden hydrogel rings bioprinted using stereolithography (**a**) CAD models of bifurcation vascular tubes (left) and the resulting hydrogel vessels visualized by their cross-section and top view (right). (**b**) Fluorescence image (bottom) and photography (top) of a cell-laden hydrogel construct. Republished with permission of Royal Society of Chemistry, from Three-dimensional fabrication of cell-laden biodegradable poly9ethylene glycol-co-depsipeptide) hydrogels by visible light stereolithography, Elomaa et al. Vol. 3, Issue 42. Copyright (2015), permission conveyed through Copyright Clearance Center, Inc. [56].

The fourth strategy, recently considered as a bioprinting technique, is the stereolithography apparatus (SLA). Its working principle is based on the selective cure of a photo-sensitive resin [113]. A light is applied on a photopolymer resin to selectively crosslink it layer-by-layer via a vector scanning, mask projection or two-photon polymerization [138]. One of the advantages of stereolithography is the processing time. Indeed, the production of a layer does not depend on the complexity or size. Hence, the processing time depends only on the thickness of the construct [139]. For biological applications the ink or biomaterial hydrogel needs to be chemically modified with a functional group that in combination with a crosslinker allow photo-crosslinking. However, the light source used for stereolithography is usually on the range of visible or UV light and, the later, has been reported to damage DNA depending on the intensity and the specific wavelength [140]. Some research has shown the possibility to customize commercially available light projectors to turn them into bioprinters of low cost, high resolution (~50 µm) and high cell viability (~85% after five days). Wang et al. printed cell-laden fibers using a home-made printer using a crosslinker in the range of green light (less harmful) and applied a water filter to reduce the temperature induced by infrared. To reduce the temperature, they applied a water filter between the resin and the light source that blocked the IR [139]. Although they did not produce a direct vascular application with their custom-built bioprinting system, the possibility to print cells with a significant resolution and a good cell viability make this technique a relevant candidate. Recently, Grigoryan et al. exploited the use of food dyes as photo-absorbers in a photo-crosslinkable hydrogel to reduce the light diffusion and increase the resolution of their constructs [141]. As a result, they could fabricate intricate structures to study the oxygenation of red blood cells (RBCs) in an alveolar-like vascular network. The bioprinted construct was perfused with RBCs but the photo-crosslinkable hydrogel itself was not loaded with cells. The important aspect of their study is the capacity to reduce the light diffusion during the process that allows to increase the resolution and the complexity of the final construct. Regarding the previous two studies exposed, a combination leading to the production of an intricate vascular model with a cell-laden resin hydrogel with a high resolution seems realistic. As an example of the potential of SLA to produce vascular constructs using a cell-laden resin, Elomaa et al. synthesized a biodegradable poly (ethylene glycol-co-depsipeptide) (PEG-*co*-PDP) macromere, photo-crosslinkable using visible light source, allowing the production cell-laden vascular networks (Figure 11) [56]. The main objective was to develop a new photo-crosslinkable biocompatible polymer to widen the repertoire of materials compatible with stereolithography. With the new synthetized polymer, they used digital light processing-based SLA to produce a vascular bifurcation in which the cells were homogeneously distributed and showed a good viability after one day of culture. Nevertheless, it seems that the resolution reached using this technique is still lower than with the previously mentioned techniques. To summarize, SLA is a promising strategy to produce vascular networks using cell-laden photo-crosslinkable materials but more materials have to be developed to this end.

## 6. Mathematical Models to Design Vasculature

The circulatory system in the human body is a complex interconnected structure spanning from large vessels with diameters on the centimeters range of the heart to micro-vessels of few microns (4–10 µm) for capillaries [142]. The vascular network can be defined as the successive division of a single vessel (mother vessel) into at least two distinct vessels (daughter vessels), named branching. Several vascular patterns are represented in the circulatory system, depending on their localization, function and scale. Even if they have their own properties and specifications, similarities can be identified. A recurrence in the patterning of the vascular morphology has been investigate from decades and it remains a challenge to develop mathematical models that can reproduce the complexity of the native intricate vascular network. A correlation with fractal models has been widely used, to represent the different vascular patterns [143,144,145,146,147,148,149,150]. Fractals corresponds to branching self-similar patterns, meaning they have the same structure at every scale of the different branches [151]. Another approach to design vascular network patterns is based on the principle of the minimum energy to transport nutrients and eliminate waste in the circulatory system and was first describe by Murray in 1926 [152,153]. From his study, several mathematical models have emerged, such as the Huo-Kassab (H-K), the Finet and the area-preservation models.

### 6.1. Fractals Trees

**Figure 12:**
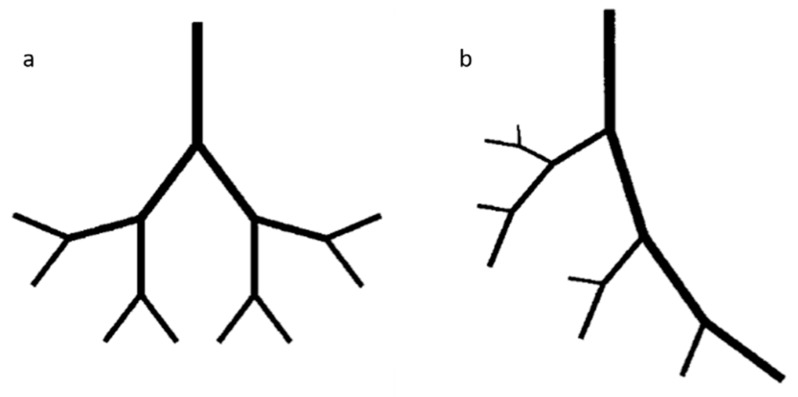
Fractal representation of a symmetrical bifurcation (**a**) and a non-symmetrical bifurcation (**b**) of a vascular tree. Reprinted from Fractal Dimensions and Multifractility in Vascular Branching, M. Zamir, J. Theor. Biol. (2001) 212, 183–190 (Issue 2), Copyright (2001), with permission from Elsevier [150].

One of the main criteria for an efficient vascular system is to fill as much as possible the space in order to distribute the flow everywhere in a tissue [150,154]. A mother vessel splits into two daughter’s vessels, and then both daughter’s vessels split, as mother vessels, into two new daughter vessels (Figure 12). Fractal trees are defined by the repetition of symmetric or asymmetric binary branching (bifurcation) and are composed of an infinite repetition of self-similar bifurcations. Thus, fractal patterns are a promising approach to design vascular networks. However, experimental studies have shown the asymmetric aspects of native vascular bifurcations [155,156] in terms of daughter’s vessels length and diameter. Moreover, within the full vascular system, the self-similarity is limited from one level of branching to another one. Hence, the native vascular system can be considered and studied as a “quasi-fractal” model for a specific segment location and scale [142,150].

### 6.2. Mathematical Model for Bifurcation

**Figure 13:**
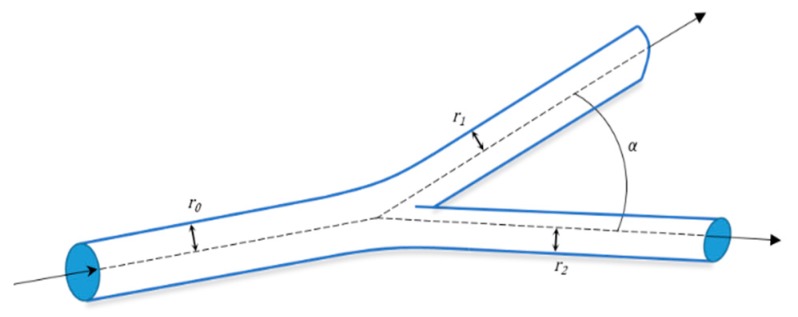
Schematic representation of a vascular bifurcation where *r*_0_, *r*_1_, and *r*_2_ are the radii of the mother and daughter vessels, respectively, and *α* is the angle between the two daughter vessels.

Murray was one of the first to study the optimization of vascular systems in 1926 [152,153,157]. He first considered the arterial system following the “principle of economy” to develop an optimally relevant model and found that two main factors were involved. Indeed, the main requirement for small vessels are related to maintaining a sufficient blood flow while the requirement for large vessels is the blood volume [152,153]. From the Poiseuille equation, he found the simplest condition to reach the maximum efficiency of blood circulation, now named as Murray’s law (Equation (1)). In Murray’s Law, *r*_0_, *r*_1_ and *r*_2_ are the radii of mother and daughters vessels, respectively, for a single branching segment (Figure 13) [157].

(1) $${\mathrm{Murray}}^{’}s\;\mathrm{law}:r_{0}{}^{3}=r_{1}{}^{3}+r_{2}{}^{3}$$

The previous law has been widely used to design and study vascularized systems [158,159]. However, new mathematical models have been established derived from Murray’s law (Equations (2)–(4)) [160].

(2) $$\mathrm{Area}-\mathrm{preservation}:r_{0}{}^{2}=r_{1}{}^{2}+r_{2}{}^{2}$$

(3) $$\mathrm{Finet}\;\mathrm{model}:r_{0}=0,678\;\left(r_{1}+r_{2}\right)$$

(4) $$H-K\;\mathrm{model}:r_{0}{}^{7/3}=r_{1}{}^{7/3}+r_{2}{}^{7/3}$$

Each model considers different limitations and constraints that lead to more accurate models for each specific application and condition. These models include extended constraints resulting in more specific and physiologically relevant conditions. The area-preservation and Finet models are based on fractal geometries, thus they are based on empirical models. The area-preservation model considers the sum of the daughter’s vessels areas equal to the mother vessel area. This assumption is refuted by observation of the native vascular anatomy. Moreover, the area-preservation model assumes a constant velocity, which is in contradiction with experimental measurements. In the case of Finet, the model has the advantage of being simple but without respecting the principle of mass conservation. Murray’s law is based on mass conservation and minimum energy hypothesis for a laminar flow corresponding to a single bifurcation segment (local) while the H-K model considers the blood flow for the entire vascular system (global). Hence, it is based on physiological models. Nevertheless, Murray’s law assumes that the Wall Shear Stress (WSS) is constant all along the vasculature, which is not corresponding to the experimental measurements of the native system [154,161].

(5) $$\mathrm{Murray}:\;\cos\left(\alpha \right)=\frac{{\left(r_{2}{}^{3}+r_{1}{}^{3}\right)}^{4/3}-r_{2}{}^{4}-r_{1}{}^{4}}{2\left(r_{2}{}^{2}\right)\left(r_{1}{}^{2}\right)}$$

(6) $$\mathrm{Finet}:\cos\left(\alpha \right)=\frac{0.678^{4}{\left(1+r_{1}/r_{2}\right)}^{4}-\left(1+{\left(r_{1}/r_{2}\right)}^{4}\right)}{2{\left(r_{1}/r_{2}\right)}^{2}}$$

(7) $$H-K:\cos\left(\alpha \right)=\frac{{\left(1+{\left(r_{1}/r_{2}\right)}^{7/3}\right)}^{12/7}-\left(1+{\left(r_{1}/r_{2}\right)}^{4}\right)}{2{\left(r_{1}/r_{2}\right)}^{2}}$$

From Equation (1), Murray developed a theoretical law to describe angles between mother and daughter vessels at a bifurcation (i.e., branching segment) while minimizing the work (Equation (5)) [157,160]. α corresponds to the angle between the two daughter vessels (Figure 12). Huo et al. modified Murray’s bifurcation angle rule developing revised versions of H-K and Finet models (Equations (6) and (7)). Then, they investigated the thrombus formation on a coronary bifurcation depending on the previous Law and models. In this study, they investigated hemodynamic parameters at bifurcation, which are the WSS and the Oscillatory Shear Index (OSI), comparing the angle between daughter vessels and the diameter ratio of the vessels depending on the different models. They exposed that increasing the WSS and decreasing the OSI at the bifurcation point prevents thrombus formation. Here, they considered Murray’s angle rule as an artificial “Y” shaped bifurcation type, while Finet assumes an artificial “T” shape bifurcation type. The H-K model corresponds to both bifurcation types depending on the defined angle (“T” shape bifurcation > 60 > “Y” shape bifurcation). The results of their study showed that both the “Y” bifurcation assumed by Murray and the “T” bifurcation by Finet present a lower WSS and higher OSI at bifurcation, which is in contradiction with experimental measurements (porcine coronary arterial tree measurement using CT scan [162]). These results questioned the hemodynamics optimality of Murray’s bifurcation angle rules [160]. Conversely, the H-K model is in agreement with the measurements of a “Y” and “T” shaped bifurcation and provides high WSS and low OSI. It seems, based on the work of Huo et al., that the H-K model should be more accurate in terms of vascular patterning related to the bifurcation angle and diameter ratio for a global vascular system, as compared to Finet’s and Murray’s. Nevertheless, it seems complicated to develop a model including all parameters and constraints related to physiological conditions. However, some of the developed models are more relevant and can be accurately applied to specific systems.

## 7. Emerging Techniques

3D bioprinting is a field in continuous evolution and development. Significant improvement has been achieved in recent years in manufacturing to produce complex 3D biological structures [163]. Innovative and emergent methods have been developed to overcome existing challenges and limitations such as unsuccessful removal of sacrificial biomaterials, poor resolution and high cell death.

**Figure 14:**
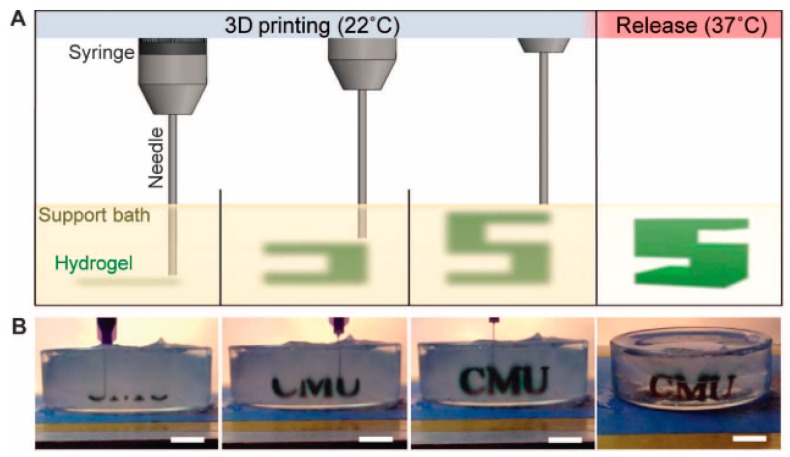
FRESH technique. Injection of hydrogel ink into the support bath at 22 °C layer-by-layer (**A**). Example of letters “CMU” printed in alginate into a gelatin support bath (Scale bar is 1 cm) (**B**). Reprinted from Reference [39], with permission from Science Advances.

A technique that has grown attention over the past years is the freeform reversible embedding of suspended hydrogels (FRESH) (Figure 14) [164]. This layer-by-layer fabrication method works in an extrusion-based modality and consists in utilizing two different hydrogels: the primary gel that is being deposited and a secondary gel that is used as support bath. An alginate ink is deposited at 20–22 °C into a thermoreversible gelatin support bath where the secondary hydrogel acts as fugitive ink. The bath provides mechanical support during bioprinting, improving resolution and bioprinting accuracy. When the fabrication is completed, the construct is extracted by melting the gelatin bath at 37 °C without damaging the deposited hydrogel. However, the deposited hydrogel must quickly be crosslinked into the bath containing calcium ions to obtain the desired pattern and avoid diffusion. FRESH was used to fabricate 3D structures of multiple shapes and dimensions [39].

FRESH allows fabricating complex and interconnected 3D structures. Femur models and vessels with bifurcations have been fabricated with high fidelity by embedding cells in the bioprinted hydrogel. In addition, this technique can also be implemented to allow multiple material deposition by dual syringe extrusion.

In a recent study, a novel microspheroid bioprinting method was developed using an airflow-assisted system [105]. This technique allowed having a spatially defined architecture inside the spheroid. A microfluidic nozzle from a PDMS chip was used to dispense alginate droplets with encapsulated cells. After bioprinting, an airflow at the outlet of the nozzle rotates the droplets giving a spiral pattern to the bioink. The final spheroid was crosslinked with CaCl_2_ solution to form a hydrogel. In this way, it is possible to control the size of the droplet, which increases with time, and to have fluids stratification with multiple cell types. In fact, the spheroid can display the spiral pattern inside, on and outside according to the airflow position, leading to different compartments. This design can be useful for cell nutrient diffusion or for modeling 3D culture environment.

### Multi-Material Bioprinting

Native biological tissues are constituted by different cells, materials and biomolecules working in synergy and leading to a particular function. By depositing a single material at a time, mimicking this complexity seems to be utopic. To overcome this issue, new techniques emerged to deposit simultaneously several bioinks. The multi-material bioprinting is possible as co-extrusion, multi-jet including side-by-side, co-axial bioprinting, core-shell bioprinting or gradient. Practically, the multi-material bioprinting consists in bioprinting more than one material in a single step. In co-extrusion set-ups the different materials are mixed in the same bioink and simultaneously extruded together through the same nozzle. This technique can be used to improve the mechanical properties of a given hydrogel [113] or to include a drug or biomolecules in order to increase cell viability [165]. Alginate hydrogels can undergo crosslinking on the presence of divalent cations such as those on a CaCl_2_ solution. Gauvin-Rossignol et al. produced a carbohydrate glass fugitive ink containing CaCl_2_ embedded in an alginate hydrogel solution in order to increase locally the crosslinking of alginate when it goes in contact with the sugar (carbohydrate glass). The fugitive ink dissolved in contact with the hydrogel. Hence, if the crosslinking time is longer than the dissolution of the fugitive ink, it leads to the loss of the imprinted vascular structure. Here, the CaCl_2_ contained in the carbohydrate glass enhanced the crosslinking locally around the fiber, increasing the stiffness of the hydrogel through its reticulation while it dissolved and avoided the collapse of the channel, leading to the retention of the alginate shape around the fugitive ink [166]. Another example is the co-deposition of cells. Indeed, cells need growth factors and signals produced most of time by other cell types. This physiologic synergy is often required for cell maturation and differentiation into specific phenotypes. Using the same approach as exposed above, the combination of several cell types in the same bioink can improve cell differentiation. To form capillary networks, ECs need growth factors such as VEGF that are secreted by fibroblasts. The co-culture of these two cell types can bring the stromal source and the growth factors needed by the ECs to maturate and self-assembly into micro-vascular networks [167].

Multi-jet bioprinting refers to the extrusion of different materials simultaneously or alternatively from different cartridges or reservoirs. Since these techniques are still evolving, there is to date, no clear classification of those. In this review, we divide these techniques in three categories: distinct-needle multi-jet (DNMJ), single-needle multi-jet (SNMJ) and intricate-needle multi-jet (INMJ). Each category allows specific material patterning to build engineered tissues.

DNMJ bioprinting combines the use of multiple bioinks deposited using separated and distinct nozzles. Since the bioinks are independent from each other, complex scaffolds with different types of hydrogels, materials and cell suspensions can be printed alternatively throughout the same scaffold [168]. Nevertheless, an important limitation is that DNMJ allows printing of only one material at time. That implies that the printing process is usually longer since it is necessary to switch the printhead to flow a different bioink [169].

**Figure 15:**
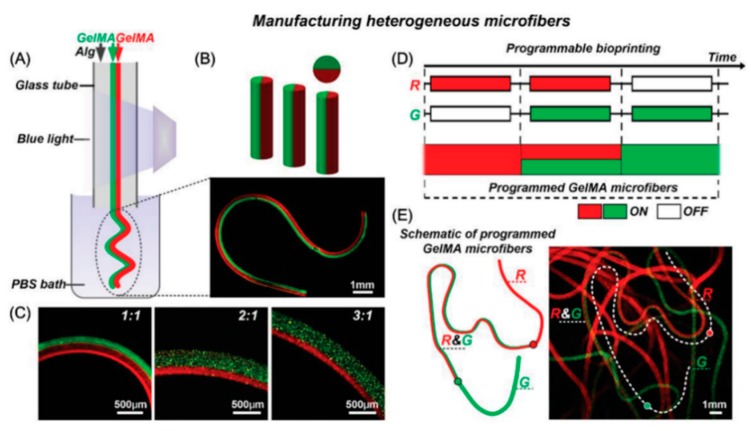
Example of SNMJ. Fabrication of the heterogeneous GelMA microfibers and 3D constructs. (**A**) Schematic illustration of the generation of GelMA microfibers with Janus structure. (**B**) Fluorescence microscopy images with two compositions GelMA microfibers. (**C**) Fluorescence microscopy images with different composition ratios (1:1, 2:1, and 3:1) of GelMA microfibers. (**D**) Schematic of the programmable bioprinting for the generation of programmed GelMA microfibers (R, red; G, green). (**E**) Programmed GelMA microfibers with controllable compositions. Panels (**A**–**C**) refer to a side-by-side SNMJ. Panels (**D**,**E**) refer to gradient formation. Reprinted from Bioprinting of Cell-Laden Microfiber: Can It Become a Standard Product?, Shao et al. Copyright (2019), with permissions from Wiley [172].

To tackle the previous exposed limitations, SNMJ bioprinting combines the use of several bioinks from separate cartridges through a unique common needle. This technology provides several possibilities such as side-by-side patterning two different materials from two different cartridges through the same nozzle (Figure 15a–c) [170,171,172]. In addition, by adapting the flow of both bioinks, it is possible to extrude simultaneously, separately or forming a gradient within in same scaffold (Figure 15d,e) [173]. The SNMJ technology is commonly used in microfluidic devices and recently bioprinters started to replace conventional nozzles by microfluidic chip printheads [174,175].

**Figure 16:**
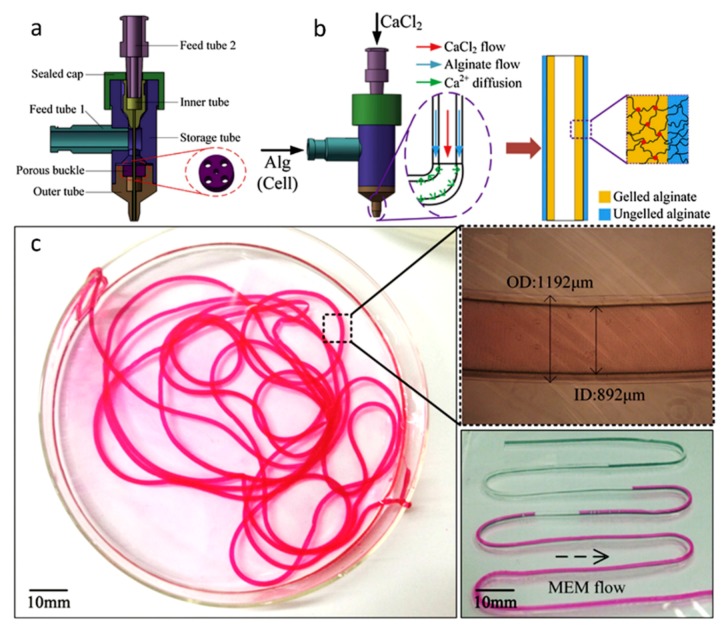
Example of INMJ. (**a**) Cross-section of a co-axial nozzle assembly model. (**b**) Schematic of the fabrication of the hollow filament (**c**) photography of a printed alginate hollow filaments (left) and inverted microscopic image of the hollow filaments (top right). Perfusion of the filament with cell culture media (bottom right). Reprinted from Coaxial nozzle-assisted 3D bioprinting with built-in microchannels for nutrients delivery, showing a co-axial needle allowing to print hollow filament using a core–shell concept. Gao et al. Copyright (2015), with permission from Elsevier [94].

More complex needles emerged on the last decade and opened the INMJ technology. This principle is mainly represented by co-axial bioprinting. The concept is to connect a needle, either with another one, in order to extrude several bioinks simultaneously. The purpose is to obtain during extrusion, several concentric layers of bioink. For example, an external layer consider as a shell using one bioink around an internal core constituted by a second bioink [165,176]. This technique can be used to crosslink the outer part of the core ink during the printing or to create tubular structures (Figure 16a,b) [94]. Gao et al. used an INMJ system in which the co-axial needle is composed by two needles (feed tubes 1 and 2), an inner and outer tube (Figure 16a) [94]. Here, feed tube 1 is used to drive a sodium alginate solution until the outer tube. A CaCl_2_ solution is extruded through feed tube 2 until the inner tube. When the two solutions are in contact, the Ca^2+^ diffuse into the sodium alginate solution, crosslinking it, and leading to the formation of an alginate filament with a hollow channel (Figure 16c).

## 8. Conclusions and Future Perspectives

Vascularization of tissue engineered constructs remains a challenge for the field that would need to be overcome before these can be successfully transplanted. Bioprinting holds a great promise to this end. Multiple bioprinting techniques have been exploited to in vitro fabricate vasculature including extrusion-based, droplet-based, laser assisted and stereolithography bioprinting, all with relative success. Within these, strategies such as sacrificial templating or multi-material bioprinting appear to be of particular interest to successfully fabricate vasculature. The formation of tubular structures imbibed within matrix hydrogels that can be perfused is nowadays feasible. However, each of these techniques and strategies require of specific materials with different properties, where viscosity, thermal transitions or biocompatibility/bioactivity play a major role. Thus, for each targeted tissue (other than vasculature) and fabrication technology, a different material and material functionalization route has to be selected.

From the formation of perfusable tubular structures to the fabrication of vasculature, there is still an important gap that has to be bridged. Blood vessels are structured on distinct layers composed of different proteins and cell types that would need to be mimicked to provide a fully functional vascular network. The main drawback of bioprinting still resides in the pressure and material crosslinking methods or temperatures that need to be used to create defined structures. Therefore, most of the studies reported so far are still based on the use of cell lines that are not translatable to clinical scenarios, but are easier to be manipulated and successfully deposited with a bioprinting set up, avoiding cell death.

Another important factor on the development of realistic vascular networks are the mathematical models used to define the network that have to convey, as it is on the native tissue, with minimal work and maximum flow. While multiple approaches have been followed, it is still not realistic to expect artificial networks below hundreds of µm in diameter. This is partly due to the limitations of the techniques and partly due to the materials themselves that make it difficult to obtain the required accuracy due to, e.g., rheological properties and swelling.

In the coming years, and for the successful development of the field, an increased knowledge on cell biology has to be gained and a substantial development of materials has to occur. While we are reaching a state of development in which the design and fabrication of tubular capillary-like networks and the endothelialization and maturation of vasculature within appears to be feasible, the formation of multi-scale vasculature and vascularized tissues are still unmet challenges. The formation of vascularized tissue engineered constructs, thus, represents the next goal of the field. To do so, multiple cells have to be combined on a spatially controlled manner and the differentiation of cell and tissue formation has to be orchestrated to timely match the growth of the target tissue and the formation of vasculature within. Moreover, many of the target tissues, such as bone, possess mechanical properties that are far from the ones of the hydrogel based bioinks formulated nowadays. Others, such as the kidney, account for a complex hierarchical structure and finely tuned functionality. Therefore, material scientist have now the challenge to develop novel bioinks that allow for a precise spatial control during the bioprinting process (allowing a spatial cell control) and account for adequate mechanical properties while being biocompatible and capable to steer tissue formation. It is also important to highlight that, thus far, most of the commonly used bioinks are based on naturally occurring materials while synthetic materials could bring plenty of possibilities to the field.

Altogether, with its advantages and disadvantages, bioprinting still appears to be the most promising technique to fabricate implantable vascularized tissue engineering constructs. However, the research carried out so far still fails to provide a clear solution to this end. The biggest challenge on the coming years will not only be the fabrication of sophisticated vascular networks, but also the combination of these with the actual engineered tissue, providing all together a mature implantable tissue that also accounts for a mature vascular network.

## Figures and Tables

**Table 1:** Summary of materials used in vascular bioprinting, techniques, cells and strengths and weaknesses of these studies.

Materials	Technique	Crosslinking	Cells	Strengths	Weakness	Reference
Naturally derived bioinks						
Agarose	Extrusion-based	Thermal	Spheroids of CHO, HUVSMCs or HSFs.	-Spatial control -Spheroid fusion -Flexible and branched structure	-Large number of spheroids required -Spatial resolution -Long time for spheroid fusion	[35]
Alginate	Drop-based	Ionic, with CaCl_2_	NIH 3T3	-Horizontal and vertical bifurcations	-Need to control process induced deformation	[36]
	Drop-based	Ionic, with CaCl_2_	HUVECs	-Diverse designs -Confluent and stable endothelium	-Simple geometries -No branched structure	[37]
	Drop-based	Ionic, with CaCl_2_	HeLa	-Small tubular construct -Tube integrity	-No complex network possible	[38]
	Gel on gel (FRESH)	Ionic, with CaCl_2_ within a gelatin support bath	n/a	-Good fidelity -Solid structure -Freeform deposition	-Need of flexible and elastic biomaterials	[39]
Collagen I	Drop-based	NaHCO_3_ Nebulization	HUVECs	-Angiogenic intravasation -Long-term stability	-Large construct -Increase of cell death with increased cell density	[40]
	Extrusion-based	Thermal	HCs, HUVECs and HLFs	-High functionality of HCs in co-culture	-Need of gelation post-printing -Need of a support material	[41]
	Laser-based	Thermal	EPCs and SCAPs	-Micro pattering of ECs -In Situ and in vivo bioprinting	-Imaging in situ	[42]
Fibrin	Drop-based	Fibrinogen-Thrombin	HMVECs	-Shape retention and integrity	-Simple pattern -No branched structure	[43]
	Drop-based	Fibrinogen-Thrombin	SMCs	-Stable tubular structure -Mimic blood vessel composition -No post-seeding with ECs	-Long process -Need of crosslinker and fibrinogen deposited simultaneously at the same location	[21]
	Laser-based	Thrombin and CaCl_2_	ECFCs and ASCs	-Space control -High cell availability -3D array	-Time consuming	[44]
Gelatin	Extrusion-based	Blended and gelled with thrombin CaCl_2_ and Na_3_P_5_O_10_	HCs and ADSCs	-Accurate control -Mature ECs derived ADSCs	-Barus effect and vertical compression of interlayer -Few peripheral ADSCs exhibit strands	[45]
	Extrusion-based	Blended and gelled with CaCl_2_	DPSCs	-Rapid release of VEGF -Tubular-like structure and spontaneous angiogenesis	-Angiogenesis only in the periphery in vivo	[46]
GelMa	Extrusion-based	Photopolymerization	HepG2 and NIH 3T3	-150–1000 µm channels -Low mass swelling	-Individually gelled template fibers -Less effective perfusion on smaller channels	[47]
	Extrusion-based	Photopolymerization	HUVECs and hMSCs	-Functionalization with VEGF -Stable capillary like structure -Early stage maturation	-Loss of mechanical properties due to degradation	[48]
	Extrusion-based and SLA	Photopolymerization	HUVECs and hMSCs	-Interconnected vascular network -Functionalization with VEGF -Capillary-like network	-Initial cell death by UV -Rounded cell morphology in static culture	[49]
Hyaluronic acid	µCOB	Photopolymerization	HUVECs and HepG2 or 10T1/2	-High cell viability -Formation of endothelial network in vitro and in vivo	-Bioink pattern not retained in vivo	[50]
	Extrusion-based	Photopolymerization	NIH 3T3, HepG2 and Int407	-Tunable mechanical properties	-Need of gelatin for cell attachment -Long UV exposure time -Difficult to remove HA-MA	[51]
Synthetic bioinks						
Pluronic^®^	Extrusion-based	Photopolymerization	n/a	-Good fidelity -Intricate design -Freeform deposition	-Need of flexible and elastic biomaterials	[52]
	Extrusion-based	Thermal (sacrificial)	HUVECs, hMSCs and HNDFs	-Long-term stability -Multicellular scaffolds -Retention of tubular structure	-Radial variation of cell phenotypes	[16]
	Extrusion-based	Thermal	HUVECs and hMSCs	-High cell viability -Good fidelity	-Need of humid environment -Channel diameter larger than 250 µm	[53]
PEG	Extrusion-based	Photopolymerization	NIH 3T3, HepG2 and Int407	-Good mechanical properties	-Low bioprintability and structural integrity of the polymer	[54]
	Extrusion-based	Photopolymerization	HUVECs and hMSCs	-Proliferation and early maturation of vascular cells -PEGTA increases mechanical strength	-PEGTA increases bioink viscosity remarkably	[55]
	SLA	Photopolymerization	HUVECs	-Visible light polymerization -Tunable degradation, swelling and stiffness with light exposure time	-Decrease in proliferation rate	[56]
